# Identification of an exosome-related signature associated with prognosis and immune infiltration in breast cancer

**DOI:** 10.1038/s41598-023-45325-7

**Published:** 2023-10-24

**Authors:** Qiaonan Guo, Kelun Pan, Pengjun Qiu, Zundong Liu, Jianpeng Chen, Jianqing Lin

**Affiliations:** 1 Department of Breast and Thyroid Surgery, The Second Affiliated Hospital of Fujian Medical University, Quanzhou, China; 2 Stem Cell Laboratory, Second Affiliated Hospital of Fujian Medical University, Quanzhou, Fujian China

**Keywords:** Risk factors, Predictive markers, Prognostic markers

## Abstract

Exosomes, nanosized vesicles, play a vital role in breast cancer (BC) occurrence, development, and drug resistance. Hence, we proceeded to study the potential prognostic value of exosome-related genes and their relationship to the immune microenvironment in BC. 121 exosome-related genes were provided by the ExoBCD database, and 7 final genes were selected to construct the prognostic signature. Besides, the expression levels of the 7 exosome-related genes were validated by the experiment in BC cell lines. Based on the signature, BC patients from the training and validation cohorts were separated into low- and high-risk groups. Subsequently, the R clusterProfiler package was applied to identify the distinct enrichment pathways between high-risk groups and low-risk groups. The relevance of the tumor immune microenvironment and exosome-related gene risk score were analyzed in BC. Eventually, the different expression levels of immune checkpoint-related genes were compared between the two risk groups. Based on the risk model, the low-risk groups were identified with a higher survival rate both in the training and validation cohorts. A better overall survival was revealed in patients with higher scores evaluated by the estimation of stromal and immune cells in malignant tumor tissues using expression (ESTIMATE) algorithm. Subsequently, BC patients with lower risk scores were indicated by higher expression levels of some immune checkpoint-related genes and immune cell infiltration. Exosomes are closely associated with the prognosis and immune cell infiltration of BC. These findings may contribute to improving immunotherapy and provide a new vision for BC treatment strategies.

## Introduction

Breast cancer is the most frequently diagnosed malignancy among women all over the word. Its incidence is increasing every year and its mortality rate is the second highest among female malignancies^1^. Thanks to the improvements in early detection and treatment, approximately 70–80% of BC patients with non-metastatic disease in early stage can be cured^2^. Despite of the multidisciplinary approaches, including surgery, chemotherapy, radiotherapy, and molecular targeted therapy, advanced BC patients with distant metastases are still considered remediless^3^. In addition, resistance to endocrine therapy or chemotherapy in BC patients also poses certain challenges to reduce the mortality of them^4^. Several problems remain elusive and sufficient evidence is lacking to fully clarify the mechanisms of breast carcinogenesis. Therefore, it is urgent for us to have a better grasp of the potential mechanisms involved in the progression of BC and the effective treatments against BC.

Recently, immune checkpoint inhibitors and biomarker-driven therapies have been validated as prospective candidates for a subset of BC treatments^5^. Substantial inflammatory cells were reported to infiltrate in BC, which were observed not only around the tumor but also abundantly in the tumor stroma^6^. Some previous studies indicated that the relationship between CD8 + T cells and immune escape was close. Besides, the infiltration of macrophages, antigen-presenting cells (APCs), CD4 + T cells, dendritic cells (DCs), and other tumor-infiltrating immune cells was significantly correlated with the BC prognosis^7–9^. Immunotherapy has recently made significant advances in the field of antitumor therapy. Based on immune modulation between the cancer cells and the tumor microenvironment (TME), immunotherapy offered clinical benefits over conventional treatments via sustained anti-tumor immune responses stimulated^10^. The two non-tumor components in the TME are mainly stromal and immune cells. Hence, the aberrant gene expression generated by epigenetic alterations in TME cells were proposed to predict the clinical outcomes of tumors^11^.

In 1985, exosomes were first reported in the incubation of sheep reticulocytes, those were produced from intracellular vesicles membrane by means of budding^12^. After that, exosomes were identified released from several cell types, including but not limited to endothelial cells, immune cells, and tumor cells^13^. As a type of homogenous membrane vesicles, exosomes can be collected in body fluids with an average size of 40–150 nm, for instance, serum, saliva, urine, and cerebrospinal fluid^14^. Later studies further demonstrated that exosomes played a vital role in intercellular communication and molecular transfer, containing cell-type specific exosomal proteins, lipids, and nucleic acids, as well^15^. In the interaction of cancer cells and their surrounding microenvironment, exosomes were involved in shaping the tumor immune responses by targeting MDSC, CAF, TAM, immune suppressive Treg cells and so on^16–18^. Recently, exosomes have been reported involved in various cancer progression^19–21^. In breast cancer, exosomes were shown to deliver mRNAs that could lead to tumor information transformation in non-tumorigenic cells^22^. In melanoma, exosomes were found to have a relationship with pre-metastatic niche formation, which could transfer MET to bone marrow progenitor cells^23^. Interestingly, several studies shown that a portion of the human epidermal growth factor receptor (HER) family was associated with exosomes in BC, gastric cancer and pancreatic cancer^19–21^. Hence, understanding the certain functions of exosomes in the TME can further provide novel sight in tumor diagnosis and treatment.

The aim of this study was to investigate the relationship between exosome-related genes and the tumor immune microenvironment (TIME) in BC. The gene profile data of BC patients were downloaded from TCGA (http://cancergenome.nih.gov/) and GEO (https://www.ncbi.nlm.nih.gov/geo/) database. After that, exosome-associated genetic data were extracted from ExoBCD database (https://exobcd.liumwei.org/) to analyze to establish an exosome risk model. Subsequently, the prognosis of BC patients was predicted according to the exosome-related risk model. Furthermore, as an entry point, the exosome-related risk score was adopted to identity the distinction in immune cell infiltration rate. Accordingly, we further explored the correlations between exosome-related risk scores and TIME, and investigated 4 genes previously reported to be associated with immune checkpoint blockade (ICB) as well^24^. This important therapy will be used to develop various interesting combination treatment strategies in the future.

## Materials and methods

### Data acquisition

RNA-sequencing expression data and clinical information of breast cancer patients were extracted from TCGA database and the corresponding information from GSE20685 was collected from GEO database as the training set and the validation set, respectively. After batch normalization, part of patients was excluded because of incomplete clinical data and an OS of less than 90 days. With complete information 828 BC samples from TCGA database were included as training set and 327 BC samples from GEO database were included as validation set for subsequent analysis. In addition, 121 exosome-related genes collected from ExoBCD database were provided in Table S1.

### Construction of a risk model

The exosome-related independent prognostic genes were screened via Cox regression univariate analysis of OS and presented by Forest plots. The differential expression genes (DEGs) between BC samples and normal breast tissue samples in training set were analyzed by edger package. The genes that met cutoff criteria of |log2fold change (FC)|> 1 and P-value < 0.05 were considered as DEGs, visualized by volcano plot. Afterward, the overlapping exosome-related genes of DEGs and prognostic genes were screened out via drawing Venn diagrams for subsequent analysis. In order to reduce redundant genes and obviate model overfitting, the least absolute shrinkage and selection operator (LASSO) Cox regression model was established to determine all independent prognostic genes^25^. The exosome-related genes expression levels were used to construct the risk score formula as: Risk score = $\mathop \sum \limits_{i = 1}^{n} \left( {Exp_{i} {*}Coe_{i} } \right)$. (N = 7, $Exp_{i}$ represented the expression level of each selected gene, and $Coe_{i}$ denoted the corresponding Cox regression coefficient.) According to the median risk score, patients with breast cancer were separated into low- and high- risk groups. Survival analyses were carried out in different risk groups by means of the “survminer” R package. Time-dependent ROC curve analyses were further performed to assess the predictor efficacy of this gene signature. As consequence, univariate and multivariate COX regression analyses were conducted to validate that the exosome-related risk score was an independent factor of prognosis for patients suffering from breast carcinoma. The hazard ratio (HR) was calculated to identify the 95% confidence interval. Hierarchical clustering was adopted to analyze the relationship between expression levels of 7 selected genes and molecular characteristics as well as clinical features, presented by heat map. After that, employing the “rms” R program, the nomogram was created with the 2-, 3-, and 5-year OS data of BC patients as well as the independent prognostic variables. The accuracy of the nomogram in 2, 3, and 5 years was evaluated using the area under the curve (AUC) and calibration plots.

### Functional enrichment analysis

Gene Ontology (GO) enrichment analyses and Kyoto Encyclopedia of Genes and Genomes (KEGG)^26–28^ pathway analyses were conducted for DEGs between high-risk groups and low-risk groups with the R clusterProfiler package. Biological process (BP), cellular component (CC), and molecular function (MF) are involved in GO terms. P values < 0.05 were considered significant in functional enrichment analysis.

### Assessment of the tumor immune microenvironment

Estimation of Stromal and Immune cells in Malignant Tumor tissues using expression (ESTIMATE) algorithm consist of Immune Score, Stromal Score and ESTIMATE Score^29^. The proportion of the immune-stromal component of TME was calculated by “estimate” R package. The respective scores implied the ratio of the corresponding compositions in the TME.

### Evaluation of immune cell type components

As a commonly used method for estimating and analyzing immune cells infiltration, CIBERSORT (http://cibersort.stanford.edu/) was used to evaluate the proportion of distinct cell subtypes from mixed cell specimens by RNA-seq expression profile. 22 marked immune cell subtypes are consist of 7 types of T cells, naive and memory B cells, plasma cells and myeloid subsets and LM22 is usually used to present the annotated gene expression signatures. Therefore, CIBERSORT was employed to calculate the ratios of 22 marked immune cell subtypes among different risk groups^30^. The assumption of immune cell types was accurate and statistics at P < 0.05 were used for further analysis. Eventually, the ratios of tumor immune infiltrating cell (TIIC) types for each tumor specimen were assessed, as well. The Wilcoxon test was carried out to characterize TIIC between tissues in different exosome-related risk groups.

### Correlations between immune checkpoint genes and exosome-related risk score

The genes reported to play significant roles in immune responses were collected. Afterwards, the GGPUBR, ggplot2, and ggExtra packages of R software were used to determine the associations of gene expression levels with different exosome-related risk scores.

### Cell culture and qRT-PCR (quantitative real-time PCR)

MDA-MB-231 cell lines and MCF 10A cell lines were collected from American Type Culture Collection (ATCC) and maintained according to the vendor’s recommendations. Briefly stated, MDA-MB-231 was maintained in Dulbecco’s modified Eagle’s medium (DMEM; Gibco BRL, USA), which includes 10% fetal bovine serum (FBS; Gibco, Grand Island, NY, USA), 1% penicillin–streptomycin, and high glucose. The special medium obtained from Procell (Wuhan, China; CM-0525) was used to culture MCF10A. In addition, the incubation conditions were set to 37 °C and 5% CO2 for all the cells. The RNAs were isolated from cultured cells using TRIzol reagent (Invitrogen, Carlsbad, CA, USA). Reverse transcription was conducted in accordance with the manufacturer’s instructions (Takara, Jiangsu, China). Subsequently, the SYBR Green method (Vazyme, Jiangsu, China) was used to assess the target genes’ expression in triplicate. The data analysis was performed by the QuantStudioTM 5 Real-Time PCR System (Thermo Fisher, MA, USA). The cycle threshold (CT) (2^−ΔΔCT^) method was employed to calculate the data. The expression levels were normalized to that of β-actin with the comparative CT method. The Table S2 lists the primers used in this study. Besides, the Human Protein Atlas database (HPA, https://www.proteinatlas.org) was employed to validate the protein expression of the genes through immunohistochemistry data.

### Statistical analysis

All statistics analysis were performed via R software (Version 4.0.5) (https://www.r-project.org/). Wilcoxon test was employed to examine the differences between variables of two groups. The survival data was assessed by Kaplan–Meier curve. The univariate and multivariate Cox regression analysis were employed to identify the independent prognostic factors. P  < 0.05 was regarded as of a statistical significance.

## Results

### Identification of 7 exosome associated DEGs in the TCGA cohort with prognostic relevance

**Figure 1:**
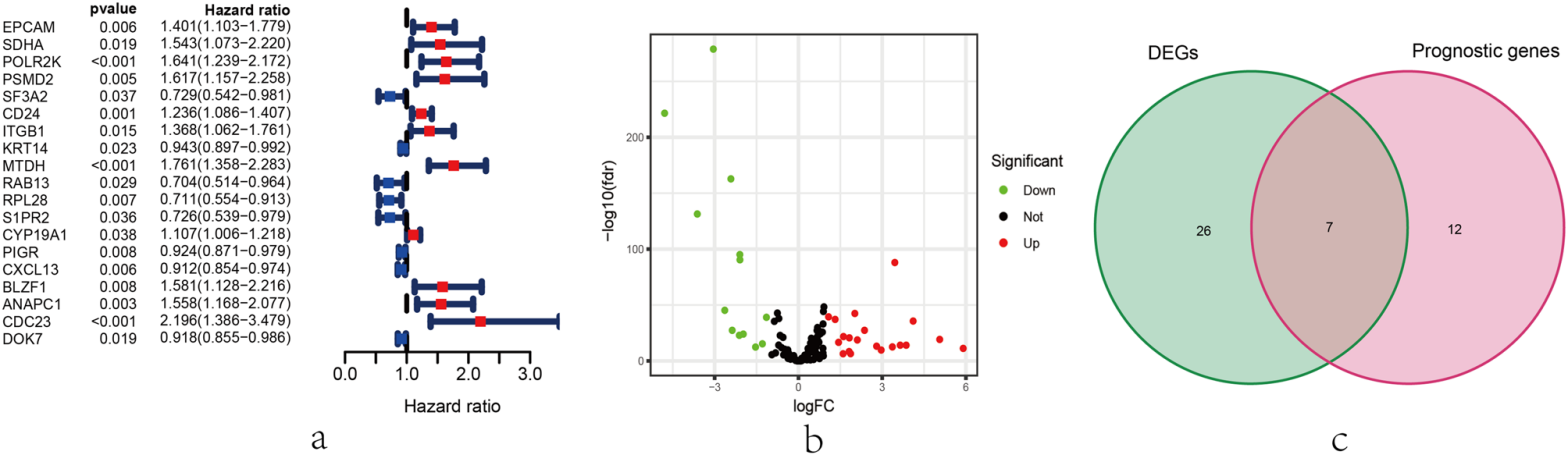
Identification of exosome-related genes differentially expressed in BC patients with prognostic significance. (**a**) Identification of exosome-related genes with prognostic significance via univariate Cox regression analysis. 19 exosome-related genes were identified associated with prognosis in BC patients (P < 0.05). (**b**) The 33 overlapping genes differently expressed in normal and tumor tissue were identified and visualized by volcano map. The green spots indicate down-regulated genes and the red spots indicate upregulated genes. (**c**) The 7 overlapping genes between DEGs and prognostic exosome-related genes were ascertained by Venn diagram.

The exosome-related gene set was downloaded from ExoBCD database, containing 121 genes participate in exosome-related regulation. Among them, 117 genes were ascertained in TCGA cohort. Subsequently, 19 exosome-related genes were identified to associate with BC patients’ OS via univariate Cox regression analysis (P  < 0.05), presented by forest plot in Fig. 1a. Afterwards, 112 normal breast tissues and 828 breast carcinoma tissues were enrolled in this study, resulting that 33 overlapping DEGs were identified by edger package (Fig. 1b). Consequently, 7 overlapping exosome-related genes were selected from DEGs and independent prognostic genes via Venn diagrams (Fig. 1c).

### Construction and validation of a gene-based prognostic model

**Figure 2:**
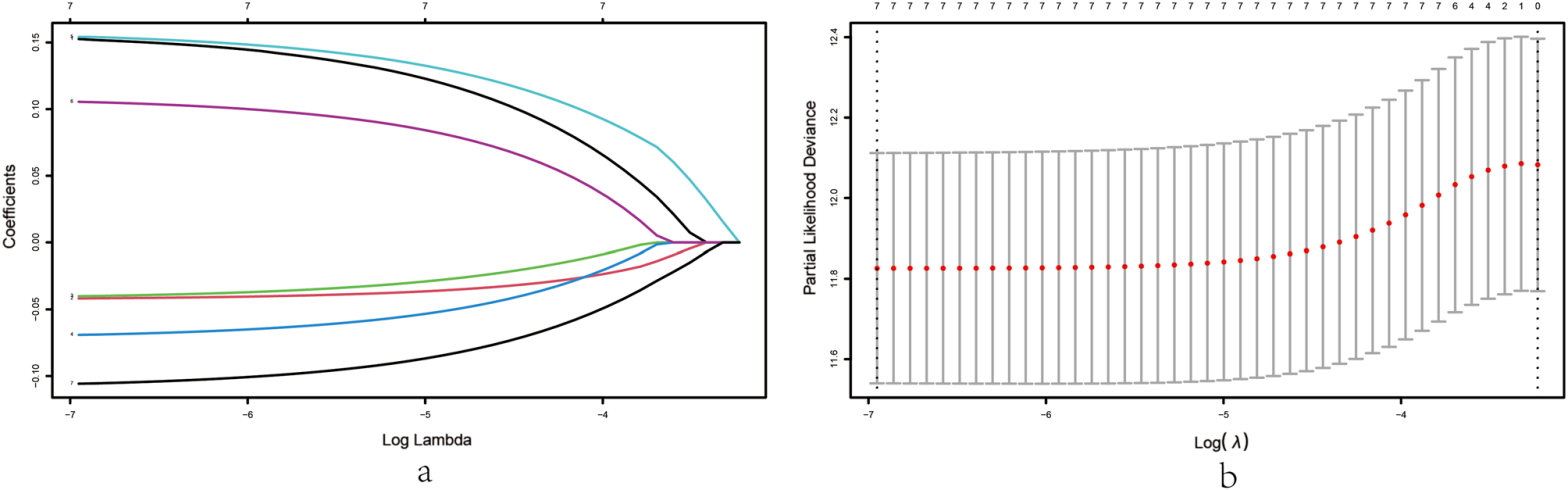
Identification of exosome-related genes in breast cancer patients with prognostic significance through LASSO Cox regression analysis. (**a**) LASSO coefficient profiles of 7 exosome-related genes with P < 0.01. (**b**) The results of the ten-fold cross-validation determined the optimal value of the penalty parameter λ.

To avoid the elimination of significant prognostic genes, 7 genes stated above were subjected to LASSO regression analysis. LASSO coefficient overview of 7 selected genes were shown in Fig. 2a and 10-fold cross-validation outcome was generated to confirm the preferred value of the penalty parameter λ (λ = 0.0009570788) (Fig. 2b). The 7 vital genes were EPCAM, PIGR, KRT14, DOK7, CD24, CYP19A1, and CXCL13. Furthermore, a prognostic risk model for evaluating BC patients’ OS was established based on the expression levels of the 7 vital genes stated above and their regression coefficients as described below: Risk score = (0.153 × expression level of EPCAM) + (− 0.042 × expression level of PIGR) + (− 0.040 × expression level of KRT14) + (− 0.069 × expression level of DOK7) + (0.154 × expression level of CD24) + (0.105 × expression level of CYP19A1) + (− 0.106 × expression level of CXCL13). Afterwards, the patients were separated into high- and low- risk groups based on the median risk score.

**Figure 3:**
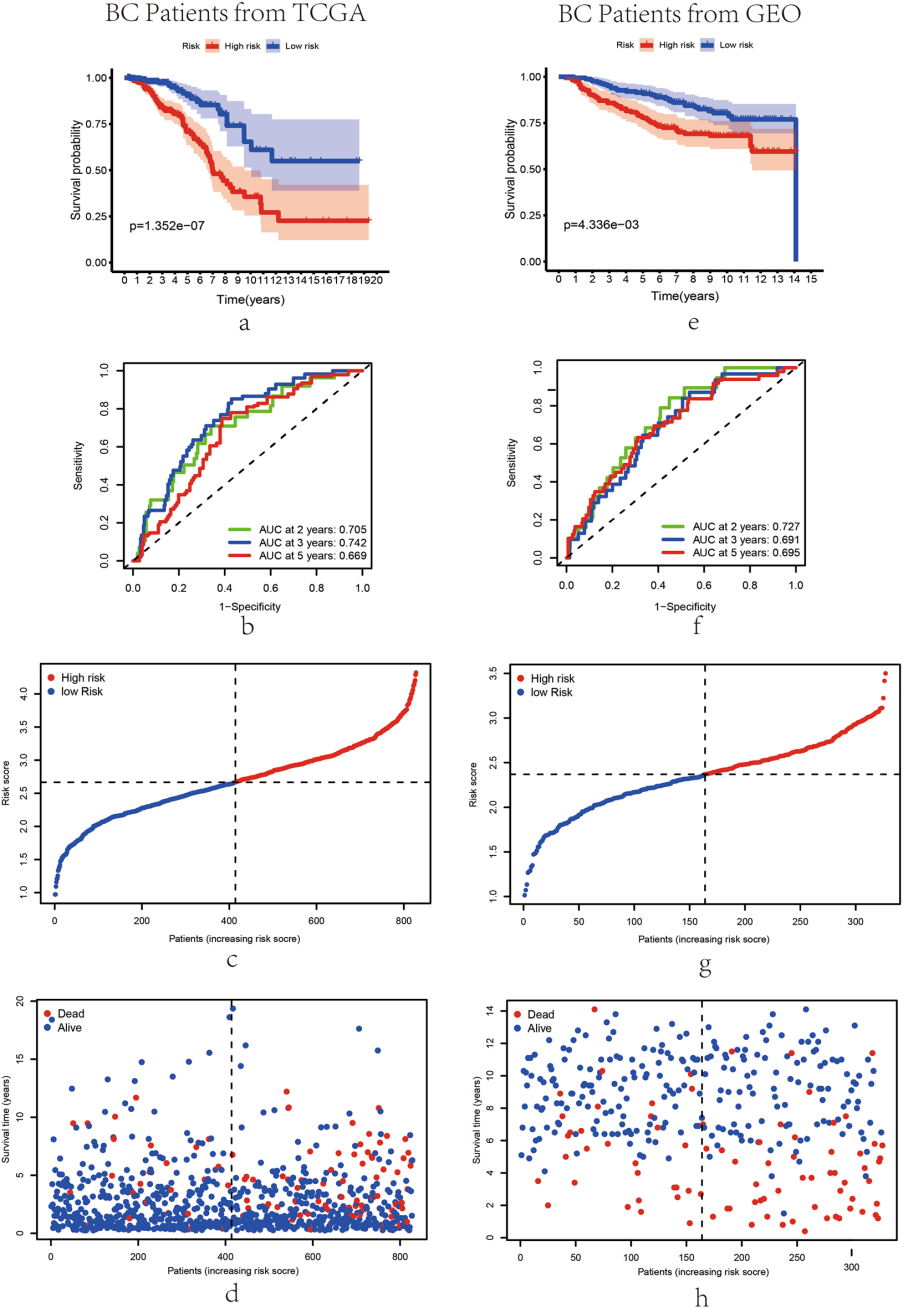
Efficacy and prognosis analysis of the exosome-associated risk model in the training and validation sets. (**a**, **e**) Kaplan–Meier survival curves for BC patients from TCGA cohort and GEO cohort, stratified based on risk scores (high vs. low); comparisons of the survival time in high-risk groups and low-risk groups with log-rank tests (P = 1.3521E−07 and P = 4.336E−03, respectively). BC patients in low-risk groups present higher survival probability. The survival curves of low-risk groups are presented as blue curves and the survival curves of high-risk groups are presented as red curves. (**b**, **f**) ROC curve analysis of model accuracy for predicting patient prognosis at 2, 3 and 5 years in the training (**b**) and validation (**f**) sets. The median value of the risk score in TCGA (**c**) and GEO (**g**) cohorts. The distributions of survival status and risk scores in TCGA (**d**) and GEO (**h**) cohort.

In Fig. 3a, the Kaplan–Meier curves suggested that patients with high-risk score are indicated worse survival rates in the training set (P < 0.01). After that, the time-dependent ROC analysis was conducted at 2, 3 and 5 years to assess the predictive efficacy of this risk model. As consequence, the prognostic features identified were verified robust efficient in predicting OS in BC patients via the AUC (AUC = 0.705, 0.742 and 0.669; at 2, 3 and 5 years, respectively, Fig. 3b). Similarly, 327 patients of GSE20685 were enrolled as the validation set and the risk score for each sample was work out based on the exosome-related signature mentioned. Figure 3e shows that patients with high-risk score are indicated worse survival rates in validation set according to the Kaplan–Meier curves (P  < 0.01). Remarkably, the risk model had been shown to have promising long-term prognostic predictive efficacy, as reflected in the time-dependent ROC analysis (AUC = 0.727, 0.691 and 0.695; at 2, 3 and 5 years, respectively, Fig. 3f). Hence, the exosome-related genes signature to evaluate BC patient’s prognosis was successfully constructed. Subsequently, based on the median risk score, the BC patients in training and validation sets were separated as high-risk groups and low-risk groups (Fig. 3c and g). As shown in Fig. 3d and h, patients with higher risk scores were manifested poorer prognosis whereas patients with lower risk scores were manifested better prognosis.

**Figure 4:**
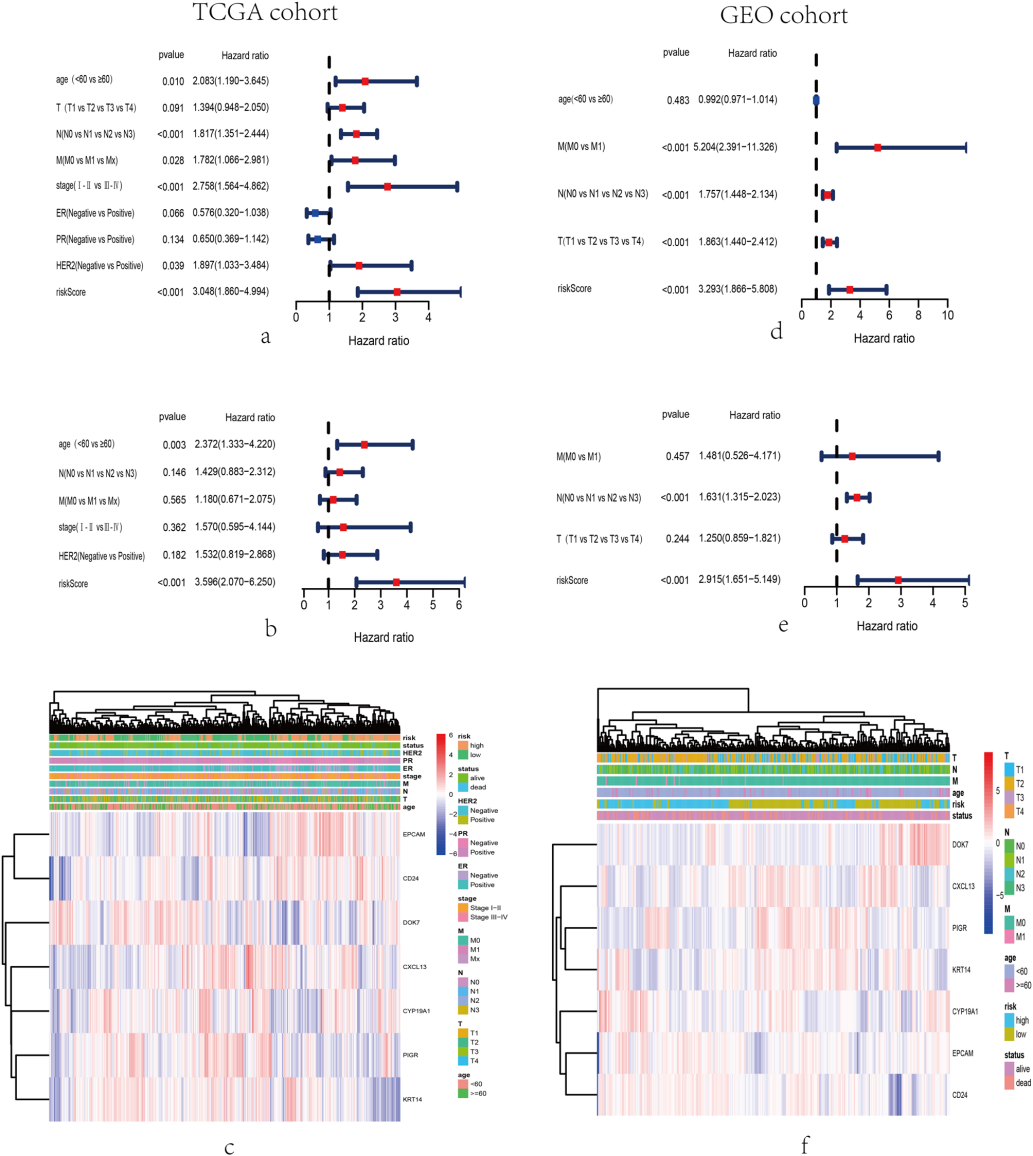
Independent prognostic value of the exosome-related risk model in the training and validation sets. The hierarchical clustering showed the associations between signature risk score, expression levels of 7 exosome-related genes, and clinical features or molecular characteristics in the training and validation datasets. (**a**) The forest plots for univariate Cox regression analysis in TCGA cohort showed that risk score (high risk vs low risk), N status (N0 vs N1 vs N2 vs N3), and AJCC stage (stage I, II vs stage III, IV) were variables related to prognostic risk. (**b**) The forest plots for multivariate Cox regression analysis in TCGA cohort showed that risk score (high risk vs low risk) were independent prognostic factors. (**d**) The forest plots for univariate Cox regression analysis in GEO cohort showed that risk score (high risk vs low risk), M status (M0 vs M1), N status (N0 vs N1 vs N2 vs N3), and T status (T1 vs T2 vs T3 vs T4) were variables related to prognostic risk. (**e**) The forest plots for multivariate Cox regression analysis in GEO cohort showed that risk score (high risk vs low risk), and N status (N0 vs N1 vs N2 vs N3) were independent prognostic factors. Heatmap illustrated the expression levels of 7 exosome-associated genes and molecular pathological characteristics or clinical features by use of hierarchical clustering in TCGA (**c**) and GEO (**f**) sets.

To further assess the efficacy of the 7-gene signature predicting the prognosis of BC patients as an independent factor, the 7-gene signature along with some covariates, for instance, tumor stage, ER, PR, HER2 and age were subjected to the univariate and multivariate Cox regression analysis. Consequently, the results revealed that the exosome-related risk score was an independent prognostic factor for BC patients in TCGA cohorts (Fig. 4a and b), also in GEO cohorts (Fig. 4d and e). Heat maps were applied to show clinical characteristics, molecular features, and distinct expression levels of 7 screened genes through hierarchical clustering in training set (Fig. 4c) and validation set (Fig. 4f). The clinicopathological characteristics of BC patients in the training and validation cohorts were presented in Table 1.

**Table 1:** Clinicopathological characteristics of BC patients from TCGA and GEO databases.

TCGA								GEO	
		High	Low	P			High	Low	P
Age	≤ 60	228	246	> 0.05	Age	≤ 60	135	147	> 0.05
	> 60	186	168			> 60	28	17	
T	T1	104	131	< 0.05	T	T1	43	58	> 0.05
	T2	243	213			T2	96	92	
	T3	45	63			T3	17	9	
	T4	22	7			T4	7	5	
N	N0	182	194	< 0.05	N	N0	61	76	> 0.05
	N1	139	149			N1	47	40	
	N2	47	41			N2	30	33	
	N3	33	28			N3	25	15	
	Nx	13	2						
M	M0	353	340	> 0.05	M	M0	157	162	> 0.05
	M1	12	8			M1	6	2	
	Mx	49	66						
Stage	I	68	86	> 0.05					
	II	228	223						
	III	91	94						
	IV	12	6						
	X	15	5						
HR	HR+	299	353	< 0.05					
	HR−	115	61						

Based on the risk score and other independent prognostic markers found in the TCGA data, the nomogram was created to forecast the 2-, 3-, and 5-year OS for BC patients (Fig. S1a). In the BC patient samples, the nomogram performed well for predicting the 2-, 3-, and 5-year OS, according to the calibration curve (Fig. S1b–d). For the 2-, 3-, and 5-year OS, the AUC of the nomogram was, respectively, 0.827, 0.827, and 0.825 (Fig. S1e). When the threshold probability is between 0 and 1, the decision curve analysis (DCA) was used to demonstrate that using this model for forecasting has benefitted BC patients (Fig. S1f).

### GO and KEGG functional enrichment analysis

**Figure 5:**
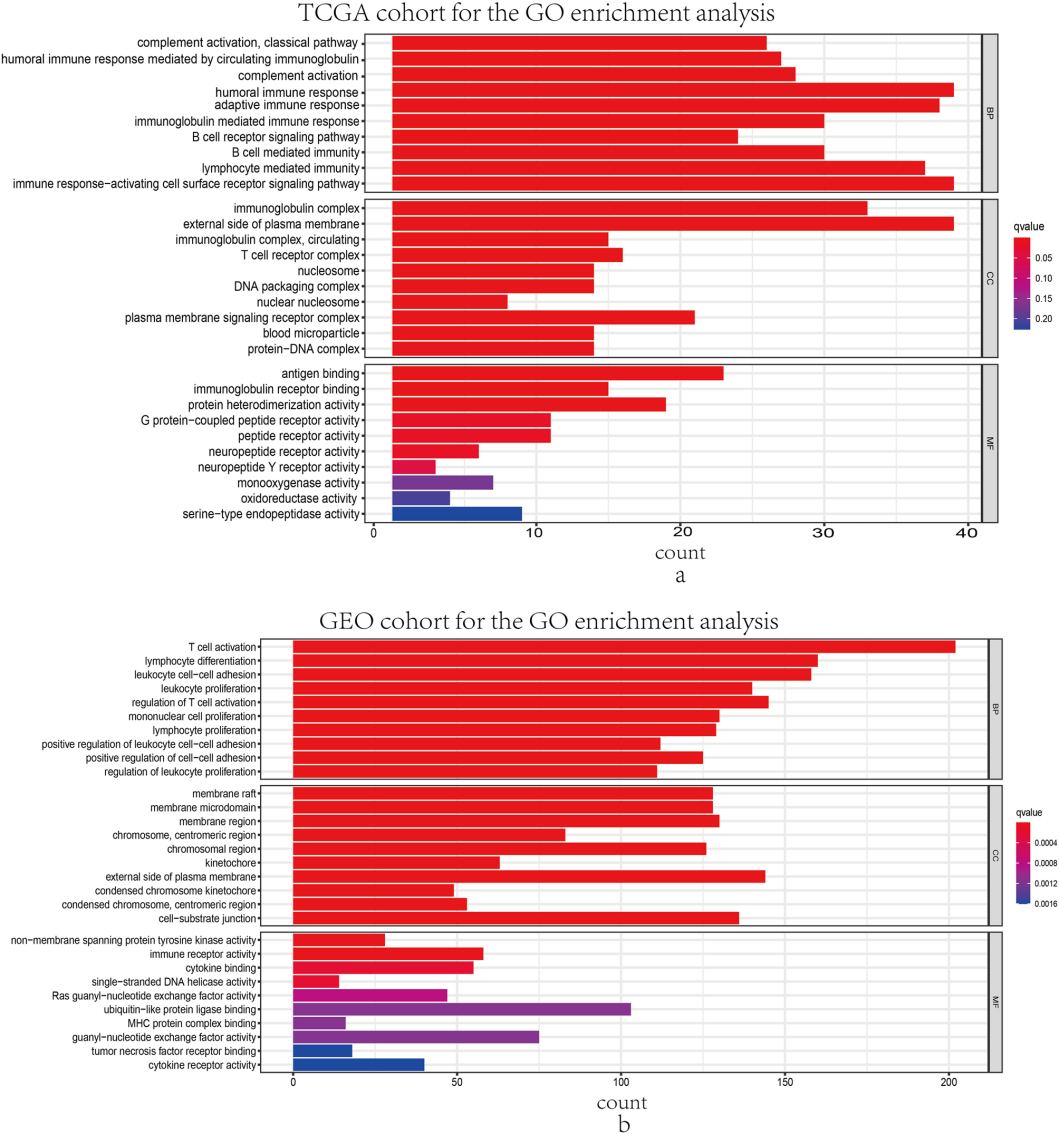
Representative findings from GO enrichment analyses in TCGA cohort and GEO cohort. The outcomes of biological process enrichment, cellular component enrichment, and molecular function enrichment in DEGs between high- and low- risk groups in TCGA database (**a**) and GEO database (**b**).

**Figure 6:**
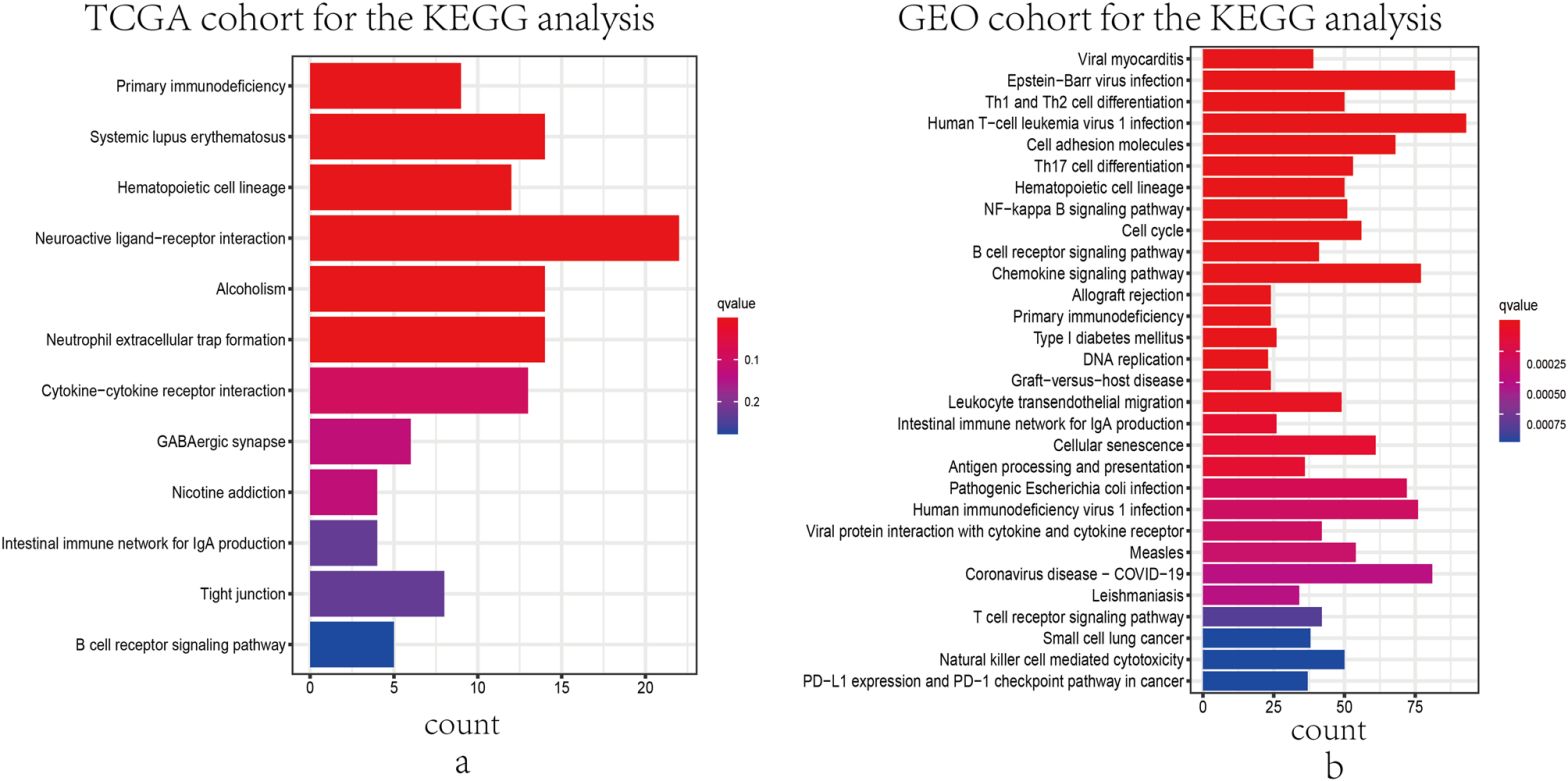
Representative findings from KEGG enrichment analyses in TCGA cohort and GEO cohort. The outcomes of KEGG pathways analyses in DEGs between high- and low- risk groups in TCGA database (**a**) and GEO database (**b**).

GO and KEGG analysis were performed in the DEGs between high and low risk groups to identify the signaling pathways and biological functions associated with exosome-related risk score. As a result, the first 30 GO terms including CC, BP and MF were displayed in Fig. 5. Additionally, 10 and 30 enriched KEGG pathways in training and validation set were manifested in Fig. 6, respectively. Most of these GO terms and the KEGG pathways were associated with immune activation as well as biological functions.

### Correlation of ESTIMATE score and 7-gene signature

**Figure 7:**
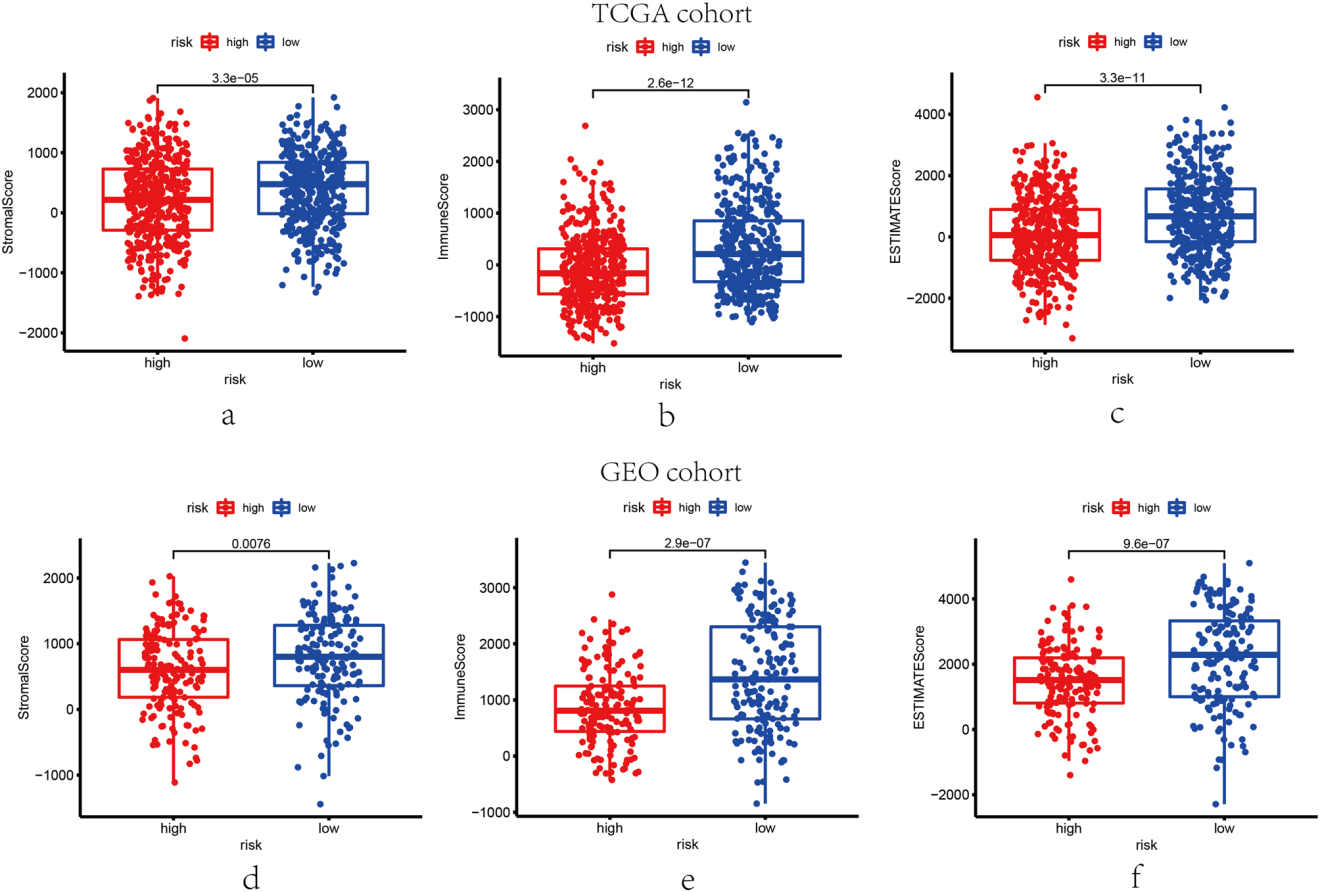
The scatter plots reveal that the distribution of stromal score, immune score and ESTIMATE score. The stromal score, immune score and ESTIMATE score were different between high- and low- risk groups in the TCGA cohort (**a**, **b** and **f**) and GEO cohort (**d**–**f**).

The ESTIMATE algorithm was applied to derive an ESTIMATE fraction for each sample, indicating the overall extent of immune infiltration and the TME landscape. As presented in Fig. 7, in both cohorts, stromal scores, immune scores, and ESTIMATE scores were proved higher in low-risk groups rather than high-risk groups (P  < 0.05). Consequently, combined with the result showed in Fig. 3a and e, the high stromal, immune and ESTIMATE scores manifested a better OS, on the other hand, the low scores were associated with the poor OS.

### Distribution of infiltrative immune cells in breast cancer

**Figure 8:**
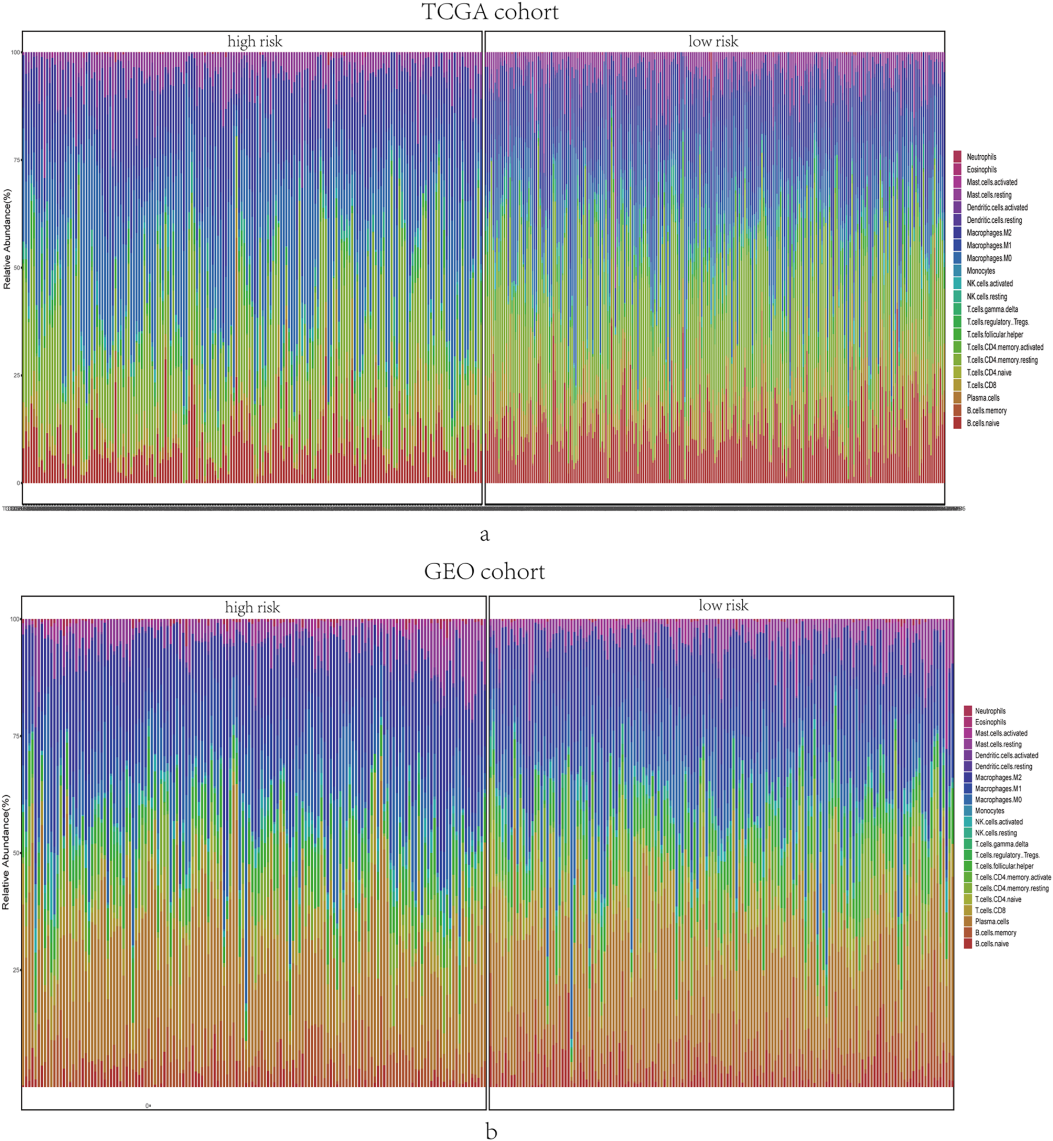
Immune infiltrations of TCGA and GEO cohort. Barplot showed the relative proportion of immune infiltration between high- and low- risk groups in TCGA cohort (**a**) and GEO cohort (**b**).

**Figure 9:**
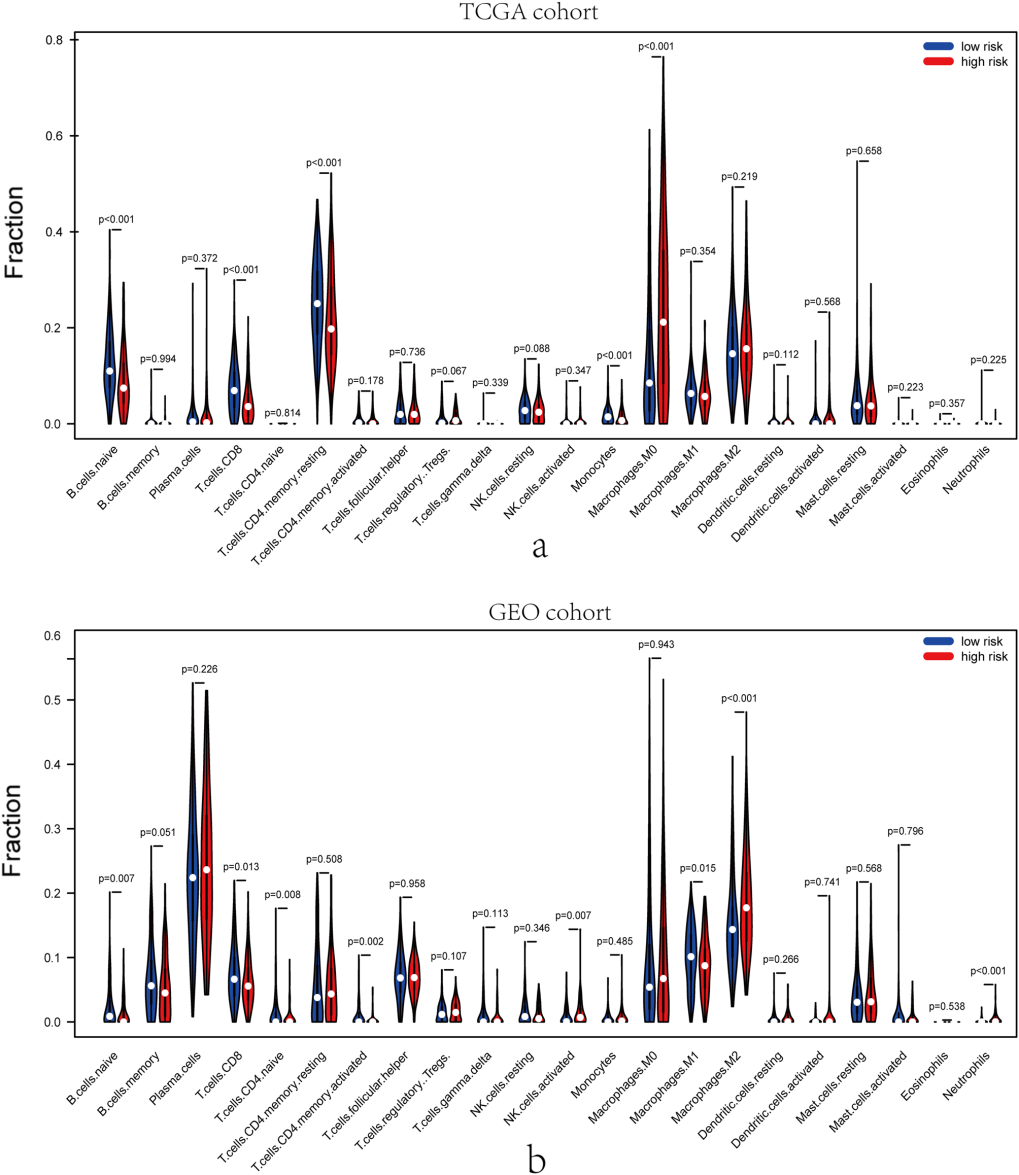
Apparent association of different immune cells between high- and low- risk groups in training and validation cohorts. The high-risk groups are marked as red and the low-risk groups are marked as blue in training (**a**) and validation (**b**) cohorts.

The functional enrichment analysis revealed that the DEGs between high-risk and low-risk groups were generally enriched in the pathways related to inflammation, immune responses, etc. Therefore, the CIBERSORT algorithm was adopted to obtain the TIIC ratios and to construct TIIC profiles. Figure 8a and b show the proportions of infiltrating immune cells in training set and validation set, respectively. As presented in Fig. 9a and b, naive B cells (P < 0.001), CD8 + T cells (P < 0.001), CD4+ resting memory T cells (P < 0.001), and Monocytes (P < 0.001) were upregulated, whereas M0 macrophages (P < 0.001) were downregulated in low-risk group of training cohort. As for validation cohort, Neutrophils (P < 0.001) were upregulated, while M2 macrophages (P < 0.001) were downregulated in low-risk group. Consequently, the study targeting exosome-related genes could be a groundbreaking finding for the immunotherapy of tumor sufferers in the future.

### The relationship between exosome-related risk model and immune checkpoint genes

**Figure 10:**
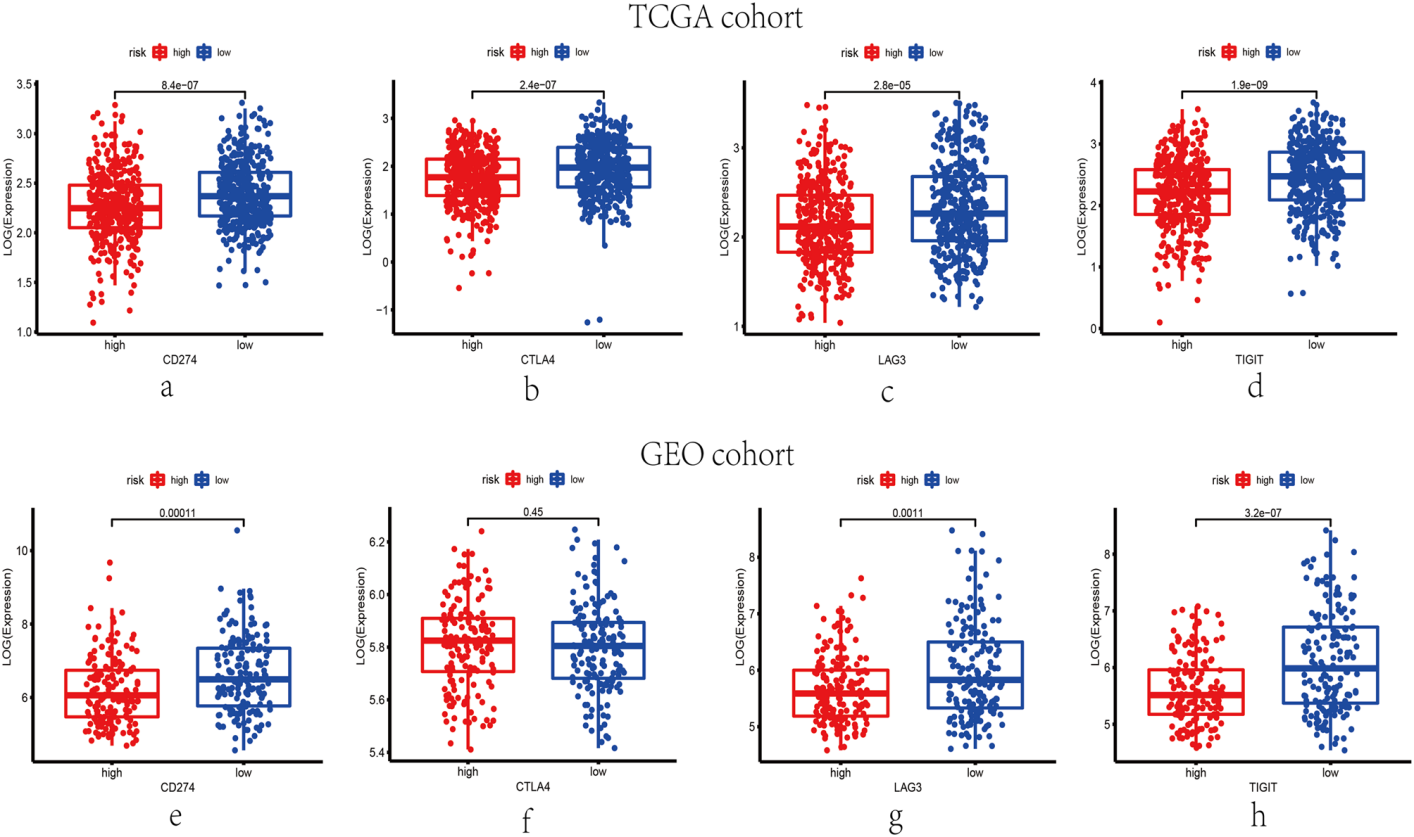
The expression levels of immune checkpoint genes in different risk groups. The expression levels of CD274 (**a**), CTLA4 (**b**), LAG3 (**c**) and TIGIT (**d**) in different risk groups of TCGA set (P < 0.001). The expression levels of CD274 (**e**), CTLA4 (**f**), LAG3 (**g**) and TIGIT (**h**) in different risk groups of GEO set (CD274, LAG3 and TIGIT: P < 0.001; CTLA4: P = 0.45).

The expression levels of immune checkpoint genes associated with the therapeutic response to immune checkpoint inhibitors were investigated. PD-L1(CD274), LAG3, CTLA4 and TIGIT were previously reported to be the targets of immune checkpoint inhibitors. As shown in Fig. 10, the expression levels of 4 mentioned genes were higher in low-risk group in TCGA cohorts. Likewise, in GEO cohorts, the expressing levels of them were upregulated in low-risk group statistically with P < 0.01, except CTLA4 (P > 0.05).

### The mRNA and protein expression of exosome-related genes involved in the risk model

**Figure 11:**
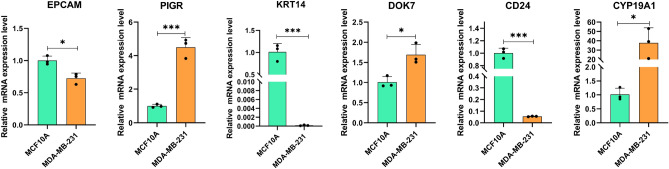
The mRNA expression levels of exosome-related genes. The mRNA expression levels of EPCAM, PIGR, KRT14, DOK7, CD24, and CYP19A1 in MCF10A (left, green) and MDA-MB-231 (right, orange). *P < 0.05, **P < 0.01, and ***P < 0.001.

The findings of the RT-qPCR assay demonstrated the degree of mRNA expression of exosome-related genes (EPCAM, PIGR, KRT14, DOK7, CD24, and CYP19A1) in normal mammary cells and BC cells. Specifically, the mRNAs of EPCAM, KRT 14 and CD24 were downregulated, while PIGR, DOK7 and CYP19A1 were significantly upregulated in MDA-MB-231 compared with MCF10A (Fig. 11). The expression of proteins encoded by PIGR, KRT14, EPCAM, DOK7, CYP19A1, CXCL13, and CD24 were shown by the immunohistochemistry staining data from the HPA database (Fig. S2).

## Discussion

BC is the highest incidence and deadliest type of carcinoma for women worldwide, presenting highly heterogeneous biological and clinical characteristics^31^. As a kind of nanosized vesicles, exosomes play vital roles in tumor development and progression. Importantly, they can regulate cell-to-cell communication in the tumor microenvironment via proteins, lipids and RNA cargo transferred^32^. Most recently, ICB therapy has made promising progress in cancer immunotherapy. In 2017, Chen and colleagues raised the idea that tumors with lower PD-L1 expression level and fewer infiltrating cytotoxic T cells were considered as immune “cold” tumors, where ICB therapy achieved only limited results. Fatally, “cold” tumors account for the majority^33^. Increasing evidence suggested that exosomes had the potentiality to work as biomarkers for a variety of malignant tumors including breast carcinoma. Previous study in pancreatic cancer identified that exosomes released from tumor contained a membrane bound protein called GPC1, considered as a sensitive and unique biomarker in early-stage disease^34^. Interestingly, a study in breast cancer indicated that the expression level of serum exosomal-annexin A2 (exo-AnxA2) could be detected higher in women with carcinoma compared with non-cancer, particularly for triple-negative breast cancer (TNBC) rather than luminal or HER2-positive breast cancer^35^. These results suggested that exosomes played an important role in BC progression and the possibility to construct a prognostic model by these exosome-related genes. In the current study, we adopted bioinformatics analysis to examine changes in the expression profiles of 121 exosome-related genes in breast cancer and the relationship with OS. Among them, 7 exosome-related genes were identified to establish a novel prognostic signature. Subsequently, the BC patients in TCGA and GEO cohorts were divided into high and low risk groups respectively according to the prognostic signature. Moreover, the DEGs between the high-risk group and the low-risk group were identified and the functional analysis were further performed, proposing that immune-related biological processes were highly enriched. Eventually, the infiltration ratios of distinct immune cells in breast cancer samples were analyzed. The results revealed that the groups at high risk of exosomes were immunologically ‘cold’, while the groups at low risk were immunologically ‘hot’.

The 7 prognostic genes associated with exosomes consist of EpCAM, PIGR, KRT14, DOK7, CD24, CYP19A1, and CXCL13. As one of the first tumor-related antigens, epithelial cell adhesion/activating molecule (EpCAM/CD326) has been reported highly overexpressed in primary and metastatic breast cancer, leading to poor prognosis^36^. Furthermore, study in hepatocellular carcinoma (HCC) suggested that tumor growth and invasion were associated with EpCAM-positive cells, which was one of the components of targeting Wnt/beta-catenin signaling pathway^37^. Cluster of differentiation 24 (CD24) is a glycosyl-phosphatidyl-inositol (GPI)-anchored glycoprotein, that has been demonstrated as a vital role in multiple areas. In cancer, CD24 is highly expressed in various tumor cells, including breast cancer cells, and associated with the growth, invasion, and migration of tumor cells^38–42^. In immunology, as a primarily costimulatory molecule, CD24 can achieve effective immunosuppression and tumor immune escape via activating a series of intracellular signal pathways and regulating multiple immune cells, for instance, T cells, B cells, macrophages, and NK cells^43^. Notably, Barkal and colleagues revealed that the macrophages enhanced their capability to engulf tumors and slowed down the development of macrophage-dependent tumors in vivo through gene knockout to blocking CD24 and Siglec-10^44^. This is consistent with our conclusion, the infiltration level of M0 macrophages is higher in high-risk group. Polymeric immunoglobulin receptor (PIGR) is a critical element of the mucosal immune system and intermediates epithelial cell transfection of immunoglobulins. And the expression level of PIGR was demonstrated decreased in nasopharyngeal carcinoma cells by Qi and colleagues, related to poor prognosis^45,46^. Several studies suggested DOK7 as a potential tumor-suppressor gene, because of the significantly low expression levels in BC tissues compared with normal tissues. The lower expression level of DOK7 was associated with the greater aggressive clinical behaviors and poorer prognosis in BC^47^. The mechanistic studies by Yue et al.^48^ illustrated that DOK7 inhibited proliferation and invasion of BC cells through PI3K/PTEN/AKT pathway. CXCL13 may be another potential protective factor for BC patients’ prognosis. Although, CXCL13 has been reported in many types of carcinomas to drive signaling pathways associated with proliferation and invasive, including PI3K/AKT pathway^49^ and Wnt/beta-catenin signaling pathway^50^, in patients with HER2-positive BC or TNBC, increased CXCL13 corresponded with better survival^51^. In addition, CXCL13 has been shown to increase B cell and T cell infiltration in multiple tumor types and associate with greater prognosis and survival^52,53^.

In our study, the results of functional enrichment analysis indicated that many GO terms and KEGG pathways were associated with biological processes and immune cells activation. In 1996, immunologists were interested in the relationship between exosomes and cells from immune system. They found that Epstein-Barr virus-transformed B lymphocytes could secrete exosomes by fusion of MVBs with the plasma membrane^54^. Furthermore, some discoveries suggested that exosomes played key roles in adaptive immune responses. Exosomes released from these kinds of cells harbor MHC class II dimers bound to antigenic peptides and the exosomes were indicated to present the MHC–peptide complexes to specific T cells. In addition, dendritic cells (DCs) in mice were reported to secrete exosomes with functional MHC class I–peptide complexes, which could improve the triggering of CD8 + T-lymphocyte-dependent immune responses^55,56^. Recently, exosomes have been shown to be involved in promoting immune responses. Studies in human pancreatic and colorectal tumours conducted by Gastpar R and his colleagues suggested that NK cells cultured with tumour-derived Hsp70-positive exosomes were induced to liberate granzyme B that activated apoptosis^57^. As the mechanisms of exosomes in antigen-specific immune responses were better understood, several studies showed that tumor-released exosomes also carried a variety of immunosuppressive molecules, such as CD8 and CD4 T lymphocytes^58,59^, NK cells^60^, regulatory T lymphocytes^61^ and myeloid cells^62^. Hence, we reasonably assumed that exosomes were closely associated with anti-tumor immunity in BC. Furtherly, The CIBERSORT algorithm was applied to derive the ratios of various kinds of tumor-infiltrating immune cells to reveal the relationship between exosomes and immune cell infiltration in BC. The result indicated that the infiltrated tumor-killing immune cells were significantly reduced in high-risk group instead of low-risk group, such as CD8 + T cells and activated NK cells. However, the immune cells that promoted tumor progression and migration, M2 macrophages and M0 macrophages, were reduced in low-risk group instead of high-risk group. As a marker of tumor progression and drug resistance, TIME was characterized by tumor inflammation promoting and tumor cells immune surveillance^63^. Recent studies in tumors observed that some immune activities were associated with exosomes. Studies in exosomes secreted from prostate cancer cells indicated that exosomes contained ligands for natural killer group 2D (NKG2D) could downregulate the expression of NKG2D on NK cells and impair the cytotoxicity of NK cells^60,64^. Besides, those exosomes secreted by HCC cells with chemotherapy treatment efficiently stimulated NK cells to product the granzyme B, hence promoted the tumoricidal function^65^. In tumor microenvironment, those tumor cell-derived exosomes could influence the DCs status. The results of experiments in vitro indicated that exosomes produced by TS/A BC cells could block the differentiation progression of DC from myeloid cells^66^. In terms of macrophages, type 1 macrophages (M1) were involved in anti-tumor immune responses by acting as antigen-presenting cells whereas type 2 macrophages (M2) acted in the pro-tumor immunity as the common phenotype of tumor-related macrophages^15^. Several studies revealed that the M2 status was correlated with the progression of tumors and poor prognostic outcomes of patients. Yang et al.^67^ reported that exosomes derived from M2 could promote BC cell growth and invasion by transferring miR-223. Additionally, neutrophils status was reported to be regulated and differentiated via the stimulation from TME, as a result, to further moderate tumor immune responses and regulate tumor progression. Bobrie et al.^68^ investigated on the value to Rab27a in exosome secreted by BC cells, consequently, exosomes were identified to induce systemic mobilization to neutrophils to facilitate tumor progression. Not only different kinds of immune cells, but also other cellular components of TME, including MSCs, endothelial cells and fibroblasts, played significant roles in tumor progression^15^. Accordingly, a conclusion was raised that the exosomes were markedly associated with the ratio of tumor-related immune cells infiltrating in BC, and the low-risk cohort tended to possess higher ratio of cytotoxic lymphocyte infiltration.

Recent research findings suggested that exosomes were able to control the core immunologic processes and regulate inflammatory response. Immune cell exosomes were identified to involve in stem cell mobilization, immunological regulation, and tissue remodeling^69^. Immunotherapy is increasingly becoming the key to BC treatment. Yang et al.^70^ found that PD-L1 could be carried by BC cell-derived exosomes and transferred to tumor cells expressed low levels of PD-L1 to blocked T cell activity. Notably, exosome-related immunotherapeutic exoPDL1 was able to be applied to develop novel drugs with minimum toxicity and considerable clinical effectiveness^69^. Furthermore, immune checkpoints, a key of facilitating tumor immunosuppression, were analyzed in different risk groups to detect the correlation between exosomes and immune checkpoint inhibitors. Stimulation of immune checkpoint targets can block tumor attack. In our study, high-risk group predicated on 7-exosome-related gene risk model was found to associate with lower expression levels of immune checkpoint genes and poor clinical outcomes. As a result, mammary tumor in high-risk group were considered immunologically “cold” and difficult to gain benefits from ICBs. However, the tumors in low-risk group were considered immunologically “hot” and more possible to get benefits from ICBs^33^. Accordingly, the exosome-related risk model had highly potential to predict the efficacy of ICBs in treatment for patients suffered from BC.

It was the first study to construct an exosome-related risk model for breast cancer based on 7 exosome-related genes by use of public databases with retrospective data. This risk model can serve as an independent prognostic factor in BC patients. In addition, some limitations in current research should be noticed. Some of the clinical data in the TCGA or GEO cohorts were incomplete and the absent data may not be random, leading to the bias in the analysis of clinical relevance. Therefore, it requires more extensive multi-center clinical validation to further back up our ideas. Although, the mRNA expression of the genes involved in the signature was validated, the confirmation at the protein expression level is very important. Besides, this prognostic model was built only from exosome-related genes and various other hotspot biomarkers were not enrolled. Actually, the correlation between the function of the exosome and some genes involved in the signature was unclear. There are a number of genes that are ubiquitous in tumor tissues, and they have regulatory pathways for tumor development other than exosomes. Hence, it is important to conduct the mechanism analysis to identify the roles that the corresponding genes play in the function of the exosome in future study. Furthermore, immunotherapy is not a mainstream modality in BC treatment strategies. But as for the BC patients who do not benefit from traditional treatment modalities, immunotherapy becomes one of the key options for them. This risk model provides a way to predict the immunotherapy efficacy of BC. And the further experimental validation of the relationship between immune cells and exosomes is needed to provide new perspectives in immunotherapy and tumor treatment.

## Conclusion

In summary, by combining bioinformatics tools and related algorithms, we identified a novel exosome-related risk model associated with immune infiltration. It can be served as a potential independent prognostic factor and bring new insights into anti-tumor immunity for breast cancer.

### Supplementary Information

Supplementary Table S1.

Supplementary Table S2.

Supplementary Information 3.

## Data Availability

The datasets analysed during the current study are available in the TCGA database (http://cancergenome.nih.gov/) and GEO database (https://www.ncbi.nlm.nih.gov/geo/) repository. The data of PCR analysis were provided in the supplementary files.
