# Supplementary material for: Identification of an exosome-related signature associated with prognosis and immune infiltration in breast cancer
Source: Sci Rep. 2023 Oct 24;13:18198. doi: 10.1038/s41598-023-45325-7 (PMC10598067; doi:10.1038/s41598-023-45325-7)
Supplement: Supplementary file 1 — Supplementary Table S1. [file 41598_2023_45325_MOESM1_ESM.pdf]

The information of 121 exosome-related genes

id	Gene Type	Gene Source	Cell Source	Catogrey	Ensemble	Chromosome/scaffold name	Gene start (bp)	Gene end (bp)	Strand	Gene description
CDC42	mRNA	pubmed	4T1	Biological interesting molecule	ENSG00000070831	1	22052627	22092946	1	cell division cycle 42 [Source:HGNC Symbol;Acc:HGNC:1736]
CP	mRNA	pubmed	4T1	Biological interesting molecule	ENSG00000047457	3	149162410	149222055	-1	ceruloplasmin [Source:HGNC Symbol;Acc:HGNC:2295]
CD47	mRNA	pubmed	MDA-MB-231, MCF-12A	Potential biomarker	ENSG00000196776	3	108043094	108091862	-1	CD47 molecule [Source:HGNC Symbol;Acc:HGNC:1682]
EEF1A1	mRNA	pubmed	MDA-MB-436, MDA-MB-231	Biological interesting molecule	ENSG00000156508	6	73515750	73523797	-1	eukaryotic translation elongation factor 1 alpha 1 [Source:HGNC Symbol;Acc:HGNC:3189]
EPCAM	mRNA	pubmed	BT474	Biological interesting molecule	ENSG00000119888	2	47345158	47387601	1	epithelial cell adhesion molecule [Source:HGNC Symbol;Acc:HGNC:11529]
ERBB2	mRNA	pubmed	BT474	Biological interesting molecule	ENSG00000141736	17	39687914	39730426	1	erb-b2 receptor tyrosine kinase 2 [Source:HGNC Symbol;Acc:HGNC:3430]
FGFR2	mRNA	pubmed	4T1	Biological interesting molecule	ENSG00000066468	10	121478334	121598458	-1	fibroblast growth factor receptor 2 [Source:HGNC Symbol;Acc:HGNC:3689]
CHEK2	mRNA	GSE93070	1833	Potential biomarker	ENSG00000183765	22	28687743	28742422	-1	checkpoint kinase 2 [Source:HGNC Symbol;Acc:HGNC:16627]
BARD1	mRNA	GSE93070	1833	Biological interesting molecule	ENSG00000138376	2	214725646	214809711	-1	BRCA1 associated RING domain 1 [Source:HGNC Symbol;Acc:HGNC:952]
TSC2	mRNA	GSE93070	1833	Biological interesting molecule	ENSG00000103197	16	2047465	2089487	1	TSC complex subunit 2 [Source:HGNC Symbol;Acc:HGNC:12363]
CASP7	mRNA	GSE93070	1833	Biological interesting molecule	ENSG00000165806	10	113679162	113730907	1	caspase 7 [Source:HGNC Symbol;Acc:HGNC:1508]
SFN	mRNA	GSE93070	1833	Potential biomarker	ENSG00000175793	1	26863138	26864457	1	stratifin [Source:HGNC Symbol;Acc:HGNC:10773]
TYR	mRNA	GSE93070	1833	Biological interesting molecule	ENSG00000077498	11	89177452	89295759	1	tyrosinase [Source:HGNC Symbol;Acc:HGNC:12442]
CCKBR	mRNA	GSE93070	1833	Biological interesting molecule	ENSG00000110148	11	6259736	6272127	1	cholecystokinin B receptor [Source:HGNC Symbol;Acc:HGNC:1571]
CYP11A1	mRNA	GSE93070	1833	Biological interesting molecule	ENSG00000140459	15	74337759	74367740	-1	cytochrome P450 family 11 subfamily A member 1 [Source:HGNC Symbol;Acc:HGNC:2590]
GATA4	mRNA	GSE93070	1833	Potential biomarker	ENSG00000136574	8	11676959	11760002	1	GATA binding protein 4 [Source:HGNC Symbol;Acc:HGNC:4173]
ABCB11	mRNA	GSE93070	1833	Biological interesting molecule	ENSG00000073734	2	168922938	169031322	-1	ATP binding cassette subfamily B member 11 [Source:HGNC Symbol;Acc:HGNC:42]
IVL	mRNA	GSE93070	1833	Potential biomarker	ENSG00000163207	1	152908545	152911886	1	involucrin [Source:HGNC Symbol;Acc:HGNC:6187]
BID	mRNA	GSE93070	1833	Biological interesting molecule	ENSG00000015475	22	17734138	17774770	-1	BH3 interacting domain death agonist [Source:HGNC Symbol;Acc:HGNC:1050]
SDHA	mRNA	GSE93070	1833	Biological interesting molecule	ENSG00000073578	5	218241	257082	1	succinate dehydrogenase complex flavoprotein subunit A [Source:HGNC Symbol;Acc:HGNC:10680]
USF1	mRNA	GSE93070	1833	Biological interesting molecule	ENSG00000158773	1	161039251	161045977	-1	upstream transcription factor 1 [Source:HGNC Symbol;Acc:HGNC:12593]
RHO	mRNA	GSE93070	1833	Biological interesting molecule	ENSG00000163914	3	129528640	129535169	1	rhodopsin [Source:HGNC Symbol;Acc:HGNC:10012]
TAC1	mRNA	GSE93070	1833	Potential biomarker	ENSG00000006128	7	97731908	97740472	1	tachykinin precursor 1 [Source:HGNC Symbol;Acc:HGNC:11517]
HLA-DQA1	mRNA	GSE93070	1833	Biological interesting molecule	ENSG00000196735	6	32628179	32647062	1	major histocompatibility complex, class II, DQ alpha 1 [Source:HGNC Symbol;Acc:HGNC:4942]
RIPK1	mRNA	GSE93070	1833	Biological interesting molecule	ENSG00000137275	6	3063991	3115187	1	receptor interacting serine/threonine kinase 1 [Source:HGNC Symbol;Acc:HGNC:10019]
CUL4A	mRNA	GSE93070	1833	Biological interesting molecule	ENSG00000139842	13	113208193	113267108	1	cullin 4A [Source:HGNC Symbol;Acc:HGNC:2554]
ABCB5	mRNA	GSE93070	1833	Biological interesting molecule	ENSG00000004846	7	20615207	20777038	1	ATP binding cassette subfamily B member 5 [Source:HGNC Symbol;Acc:HGNC:46]
ADCYAP1	mRNA	GSE93070	1833	Biological interesting molecule	ENSG00000141433	18	904943	912172	1	adenylate cyclase activating polypeptide 1 [Source:HGNC Symbol;Acc:HGNC:241]
FZD5	mRNA	GSE93070	1833	Biological interesting molecule	ENSG00000163251	2	207762586	207769563	-1	frizzled class receptor 5 [Source:HGNC Symbol;Acc:HGNC:4043]
RANBP1	mRNA	GSE93070	1833	Biological interesting molecule	ENSG00000099901	22	20115938	20127357	1	RAN binding protein 1 [Source:HGNC Symbol;Acc:HGNC:9847]
CASC3	mRNA	GSE93070	1833	Biological interesting molecule	ENSG00000108349	17	40140318	40172171	1	CASC3 exon junction complex subunit [Source:HGNC Symbol;Acc:HGNC:17040]
GPBAR1	mRNA	GSE93070	1833	Biological interesting molecule	ENSG00000179921	2	218259496	218263859	1	G protein-coupled bile acid receptor 1 [Source:HGNC Symbol;Acc:HGNC:19680]
PSMC5	mRNA	GSE93070	1833	Biological interesting molecule	ENSG00000087191	17	63827152	63832026	1	proteasome 26S subunit, ATPase 5 [Source:HGNC Symbol;Acc:HGNC:9552]
POLR2K	mRNA	GSE93070	1833	Biological interesting molecule	ENSG00000147669	8	100150584	100154002	1	RNA polymerase II subunit K [Source:HGNC Symbol;Acc:HGNC:9198]
PLCB4	mRNA	GSE93070	1833	Biological interesting molecule	ENSG00000101333	20	9068763	9481242	1	phospholipase C beta 4 [Source:HGNC Symbol;Acc:HGNC:9059]
DOK1	mRNA	GSE93070	1833	Biological interesting molecule	ENSG00000115325	2	74549026	74557554	1	docking protein 1 [Source:HGNC Symbol;Acc:HGNC:2990]
SF3B4	mRNA	GSE93070	1833	Biological interesting molecule	ENSG00000143368	1	149923317	149928344	-1	splicing factor 3b subunit 4 [Source:HGNC Symbol;Acc:HGNC:10771]
GTF2I	mRNA	GSE93070	1833	Potential biomarker	ENSG00000263001	7	74657667	74760692	1	general transcription factor IIi [Source:HGNC Symbol;Acc:HGNC:4659]
NOC2L	mRNA	GSE93070	1833	Biological interesting molecule	ENSG00000188976	1	944204	959309	-1	NOC2 like nucleolar associated transcriptional repressor [Source:HGNC Symbol;Acc:HGNC:24517]
MEF2A	mRNA	GSE93070	1833	Biological interesting molecule	ENSG00000068305	15	99565417	99716466	1	myocyte enhancer factor 2A [Source:HGNC Symbol;Acc:HGNC:6993]
CYC1	mRNA	GSE93070	1833	Potential biomarker	ENSG00000179091	8	144095027	144097525	1	cytochrome c1 [Source:HGNC Symbol;Acc:HGNC:2579]
MRPL21	mRNA	GSE93070	1833	Biological interesting molecule	ENSG00000197345	11	68891276	68903835	-1	mitochondrial ribosomal protein L21 [Source:HGNC Symbol;Acc:HGNC:14479]
MRPL4	mRNA	GSE93070	1833	Biological interesting molecule	ENSG00000105364	19	10251901	10260045	1	mitochondrial ribosomal protein L4 [Source:HGNC Symbol;Acc:HGNC:14276]
BDKRB2	mRNA	GSE93070	1833	Biological interesting molecule	ENSG00000168398	14	96204679	96244166	1	bradykinin receptor B2 [Source:HGNC Symbol;Acc:HGNC:1030]
PIK3R5	mRNA	GSE93070	1833	Biological interesting molecule	ENSG00000141506	17	8878911	8965712	-1	phosphoinositide-3-kinase regulatory subunit 5 [Source:HGNC Symbol;Acc:HGNC:30035]
PRPF3	mRNA	GSE93070	1833	Biological interesting molecule	ENSG00000117360	1	150321476	150353195	1	pre-mRNA processing factor 3 [Source:HGNC Symbol;Acc:HGNC:17348]
PSMD2	mRNA	GSE93070	1833	Potential biomarker	ENSG00000175166	3	184298709	184309054	1	proteasome 26S subunit, non-ATPase 2 [Source:HGNC Symbol;Acc:HGNC:9559]
SNRPA1	mRNA	GSE93070	1833	Biological interesting molecule	ENSG00000131876	15	101281510	101295282	-1	small nuclear ribonucleoprotein polypeptide A' [Source:HGNC Symbol;Acc:HGNC:11152]
RPS10	mRNA	GSE93070	1833	Biological interesting molecule	ENSG00000124614	6	34417454	34426125	-1	ribosomal protein S10 [Source:HGNC Symbol;Acc:HGNC:10383]
ADRA1B	mRNA	GSE93070	1833	Biological interesting molecule	ENSG00000170214	5	159865080	159972544	1	adrenoceptor alpha 1B [Source:HGNC Symbol;Acc:HGNC:278]
TRIOBP	mRNA	GSE93070	1833	Biological interesting molecule	ENSG00000100106	22	37696988	37776556	1	TRIO and F-actin binding protein [Source:HGNC Symbol;Acc:HGNC:17009]
RPL26L1	mRNA	GSE93070	1833	Biological interesting molecule	ENSG00000037241	5	172958729	172969771	1	ribosomal protein L26 like 1 [Source:HGNC Symbol;Acc:HGNC:17050]
FAM162A	mRNA	GSE93070	1833	Biological interesting molecule	ENSG00000114023	3	122384176	122412334	1	family with sequence similarity 162 member A [Source:HGNC Symbol;Acc:HGNC:17865]
SF3A2	mRNA	GSE93070	1833	Biological interesting molecule	ENSG00000104897	19	2236504	2248679	1	splicing factor 3a subunit 2 [Source:HGNC Symbol;Acc:HGNC:10766]
HER2	mRNA	pubmed	MCF7, MDA-MB-231	Potential biomarker	ENSG00000141736	17	39687914	39730426	1	erb-b2 receptor tyrosine kinase 2 [Source:HGNC Symbol;Acc:HGNC:3430]
CD82	mRNA	pubmed		Potential biomarker	ENSG00000085117	11	44564427	44620358	1	CD82 molecule [Source:HGNC Symbol;Acc:HGNC:6210]
CD24	mRNA	pubmed		Potential biomarker	ENSG00000272398	6	106969831	106975627	-1	CD24 molecule [Source:HGNC Symbol;Acc:HGNC:1645]
Del-1	mRNA	pubmed		Potential biomarker	ENSG00000164176	5	83940554	84384793	-1	EGF like repeats and discoidin domains 3 [Source:HGNC Symbol;Acc:HGNC:3173]
Dicer	mRNA	pubmed	MCF7, 4T1, MDA-MB-231	Biological interesting molecule	ENSG00000100697	14	95086228	95158010	-1	dicer 1, ribonuclease III [Source:HGNC Symbol;Acc:HGNC:17098]
POSTN	mRNA	pubmed	MCF7, MDA-MB-231	Potential biomarker	ENSG00000133110	13	37562583	37598844	-1	periostin [Source:HGNC Symbol;Acc:HGNC:16953]
FGFR3	mRNA	pubmed	4T1	Biological interesting molecule	ENSG00000068078	4	1793293	1808872	1	fibroblast growth factor receptor 3 [Source:HGNC Symbol;Acc:HGNC:3690]
FIBP	mRNA	pubmed	4T1	Biological interesting molecule	ENSG00000172500	11	65883741	65888539	-1	FGF1 intracellular binding protein [Source:HGNC Symbol;Acc:HGNC:3705]
FRS2	mRNA	pubmed	4T1	Biological interesting molecule	ENSG00000166225	12	69470349	69579789	1	fibroblast growth factor receptor substrate 2 [Source:HGNC Symbol;Acc:HGNC:16971]
FTH1	mRNA	pubmed	MDA-MB-436, MDA-MB-231	Biological interesting molecule	ENSG00000167996	11	61959718	61967660	-1	ferritin heavy chain 1 [Source:HGNC Symbol;Acc:HGNC:3976]
FTL	mRNA	pubmed	MDA-MB-436, MDA-MB-231	Biological interesting molecule	ENSG00000087086	19	48965301	48966878	1	ferritin light chain [Source:HGNC Symbol;Acc:HGNC:3999]
GSTP1	mRNA	pubmed	MCF7	Biological interesting molecule	ENSG00000084207	11	67583595	67586656	1	glutathione S-transferase pi 1 [Source:HGNC Symbol;Acc:HGNC:4638]
HOXC6	mRNA	pubmed	MDA-MB-231, T47D	Biological interesting molecule	ENSG00000197757	12	53990624	54030823	1	homeobox C6 [Source:HGNC Symbol;Acc:HGNC:5128]
HTR7	mRNA	pubmed	MDA-MB-231, T47D	Biological interesting molecule	ENSG00000148680	10	90740823	90857698	-1	5-hydroxytryptamine receptor 7 [Source:HGNC Symbol;Acc:HGNC:5302]
IGF1R	mRNA	pubmed	4T1	Biological interesting molecule	ENSG00000140443	15	98648971	98964530	1	insulin like growth factor 1 receptor [Source:HGNC Symbol;Acc:HGNC:5465]
IL1RAP	mRNA	pubmed	4T1	Biological interesting molecule	ENSG00000196083	3	190514051	190659750	1	interleukin 1 receptor accessory protein [Source:HGNC Symbol;Acc:HGNC:5995]
ITGB1	mRNA	pubmed	4T1	Biological interesting molecule	ENSG00000150093	10	32900319	33005792	-1	integrin subunit beta 1 [Source:HGNC Symbol;Acc:HGNC:6153]
KISS1R	mRNA	pubmed	MDA-MB-231, T47D	Biological interesting molecule	ENSG00000116014	19	917287	921015	1	KISS1 receptor [Source:HGNC Symbol;Acc:HGNC:4510]
KRT14	mRNA	pubmed	4T1	Biological interesting molecule	ENSG00000186847	17	41582279	41586921	-1	keratin 14 [Source:HGNC Symbol;Acc:HGNC:6416]
LDHA	mRNA	pubmed	4T1	Biological interesting molecule	ENSG00000134333	11	18394388	18408425	1	lactate dehydrogenase A [Source:HGNC Symbol;Acc:HGNC:6535]
MTDH	mRNA	pubmed	4T1	Biological interesting molecule	ENSG00000147649	8	97644179	97728770	1	metadherin [Source:HGNC Symbol;Acc:HGNC:29608]
NANOG	mRNA	pubmed	MDA-MB-231, T47D	Biological interesting molecule	ENSG00000111704	12	7787794	7799141	1	Nanog homeobox [Source:HGNC Symbol;Acc:HGNC:20857]
NEUROD1	mRNA	pubmed	MDA-MB-231, T47D	Biological interesting molecule	ENSG00000162992	2	181673088	181680876	-1	neuronal differentiation 1 [Source:HGNC Symbol;Acc:HGNC:7762]
Plau	mRNA	pubmed	4T1	Biological interesting molecule	ENSG00000122861	10	73909177	73917497	1	plasminogen activator, urokinase [Source:HGNC Symbol;Acc:HGNC:9052]
RAB13	mRNA	pubmed	MDA-MB-436, MDA-MB-231	Biological interesting molecule	ENSG00000143545	1	153981617	153986358	-1	RAB13, member RAS oncogene family [Source:HGNC Symbol;Acc:HGNC:9762]
RPL28	mRNA	pubmed	MDA-MB-436, MDA-MB-231	Biological interesting molecule	ENSG00000108107	19	55385345	55403250	1	ribosomal protein L28 [Source:HGNC Symbol;Acc:HGNC:10330]
S1PR2	mRNA	pubmed	MDA-MB-231	Biological interesting molecule	ENSG00000267534	19	10221433	10231331	-1	sphingosine-1-phosphate receptor 2 [Source:HGNC Symbol;Acc:HGNC:3169]
TGFBR1	mRNA	pubmed	4T1	Biological interesting molecule	ENSG00000106799	9	99104038	99154192	1	transforming growth factor beta receptor 1 [Source:HGNC Symbol;Acc:HGNC:11772]
TRPC5	mRNA	pubmed		Potential biomarker	ENSG00000072315	X	111774315	112082776	-1	transient receptor potential cation channel subfamily C member 5 [Source:HGNC Symbol;Acc:HGNC:12[illegible]
TSG101	mRNA	pubmed	BT474	Biological interesting molecule	ENSG00000074319	11	18468336	18527232	-1	tumor susceptibility 101 [Source:HGNC Symbol;Acc:HGNC:15971]
UCHL1	mRNA	pubmed	MCF7	Biological interesting molecule	ENSG00000154277	4	41256413	41268455	1	ubiquitin C-terminal hydrolase L1 [Source:HGNC Symbol;Acc:HGNC:12513]
ADAM10	mRNA	pubmed		Biological interesting molecule	ENSG00000137845	15	58588809	58749791	-1	ADAM metallopeptidase domain 10 [Source:HGNC Symbol;Acc:HGNC:188]
CYP19A1	mRNA	GSE93070	1833	Biological interesting molecule	ENSG00000137869	15	51208057	51338610	-1	cytochrome P450 family 19 subfamily A member 1 [Source:HGNC Symbol;Acc:HGNC:2594]
CTSB	mRNA	GSE93070	1833	Biological interesting molecule	ENSG00000164733	8	11842524	11869448	-1	cathepsin B [Source:HGNC Symbol;Acc:HGNC:2527]
ELK1	mRNA	GSE93070	1833	Biological interesting molecule	ENSG00000126767	X	47635521	47650604	-1	ELK1, ETS transcription factor [Source:HGNC Symbol;Acc:HGNC:3321]
PIGR	mRNA	GSE93070	1833	Biological interesting molecule	ENSG00000162896	1	206928518	206946466	-1	polymeric immunoglobulin receptor [Source:HGNC Symbol;Acc:HGNC:8968]
FGF9	mRNA	GSE93070	1833	Biological interesting molecule	ENSG00000102678	13	21671383	21704498	1	fibroblast growth factor 9 [Source:HGNC Symbol;Acc:HGNC:3687]
DUSP1	mRNA	GSE93070	1833	Biological interesting molecule	ENSG00000120129	5	172768090	172771195	-1	dual specificity phosphatase 1 [Source:HGNC Symbol;Acc:HGNC:3064]
MRPS30	mRNA	GSE93070	1833	Biological interesting molecule	ENSG00000112996	5	44808925	44820428	1	mitochondrial ribosomal protein S30 [Source:HGNC Symbol;Acc:HGNC:8769]
DMRT1	mRNA	GSE93070	1833	Biological interesting molecule	ENSG00000137090	9	841690	969090	1	doublesex and mab-3 related transcription factor 1 [Source:HGNC Symbol;Acc:HGNC:2934]
CXCL13	mRNA	GSE93070	1833	Biological interesting molecule	ENSG00000156234	4	77511753	77611834	1	C-X-C motif chemokine ligand 13 [Source:HGNC Symbol;Acc:HGNC:10639]
BLZF1	mRNA	GSE93070	1833	Biological interesting molecule	ENSG00000117475	1	169367970	169396540	1	basic leucine zipper nuclear factor 1 [Source:HGNC Symbol;Acc:HGNC:1065]
ANAPC1	mRNA	GSE93070	1833	Biological interesting molecule	ENSG00000153107	2	111611639	111884690	-1	anaphase promoting complex subunit 1 [Source:HGNC Symbol;Acc:HGNC:19988]
ALDH9A1	mRNA	GSE93070	1833	Biological interesting molecule	ENSG00000143149	1	165662216	165698863	-1	aldehyde dehydrogenase 9 family member A1 [Source:HGNC Symbol;Acc:HGNC:412]
ENPP1	mRNA	GSE93070	1833	Biological interesting molecule	ENSG00000197594	6	131808016	131895155	1	ectonucleotide pyrophosphatase/phosphodiesterase 1 [Source:HGNC Symbol;Acc:HGNC:3356]
CDC23	mRNA	GSE93070	1833	Biological interesting molecule	ENSG00000094880	5	138187648	138213343	-1	cell division cycle 23 [Source:HGNC Symbol;Acc:HGNC:1724]
ARF6	mRNA	GSE93070	1833	Biological interesting molecule	ENSG00000165527	14	49893092	49897054	1	ADP ribosylation factor 6 [Source:HGNC Symbol;Acc:HGNC:659]
HDC	mRNA	GSE93070	1833	Biological interesting molecule	ENSG00000140287	15	50241947	50266026	-1	histidine decarboxylase [Source:HGNC Symbol;Acc:HGNC:4855]
CSTB	mRNA	GSE93070	1833	Biological interesting molecule	ENSG00000160213	21	43772511	43776445	-1	cystatin B [Source:HGNC Symbol;Acc:HGNC:2482]
POU4F1	mRNA	GSE93070	1833	Biological interesting molecule	ENSG00000152192	13	78598362	78603560	-1	POU class 4 homeobox 1 [Source:HGNC Symbol;Acc:HGNC:9218]
TLR5	mRNA	GSE93070	1833	Biological interesting molecule	ENSG00000187554	1	223109406	223143282	-1	toll like receptor 5 [Source:HGNC Symbol;Acc:HGNC:11851]
ABCC9	mRNA	GSE93070	1833	Biological interesting molecule	ENSG00000069431	12	21797401	21942529	-1	ATP binding cassette subfamily C member 9 [Source:HGNC Symbol;Acc:HGNC:60]
RBM10	mRNA	GSE93070	1833	Biological interesting molecule	ENSG00000182872	X	47144869	47186813	1	RNA binding motif protein 10 [Source:HGNC Symbol;Acc:HGNC:9896]
DTX3L	mRNA	GSE93070	1833	Biological interesting molecule	ENSG00000163840	3	122564238	122575203	1	deltex E3 ubiquitin ligase 3L [Source:HGNC Symbol;Acc:HGNC:30323]
ADH1A	mRNA	GSE93070	1833	Biological interesting molecule	ENSG00000187758	4	99276367	99291028	-1	alcohol dehydrogenase 1A (class I), alpha polypeptide [Source:HGNC Symbol;Acc:HGNC:249]
HEXA	mRNA	GSE93070	1833	Biological interesting molecule	ENSG00000213614	15	72340919	72376476	-1	hexosaminidase subunit alpha [Source:HGNC Symbol;Acc:HGNC:4878]
MRPL15	mRNA	GSE93070	1833	Biological interesting molecule	ENSG00000137547	8	54135210	54147901	1	mitochondrial ribosomal protein L15 [Source:HGNC Symbol;Acc:HGNC:14054]
ITGB7	mRNA	GSE93070	1833	Biological interesting molecule	ENSG00000139626	12	53191318	53207307	-1	integrin subunit beta 7 [Source:HGNC Symbol;Acc:HGNC:6162]
DCT	mRNA	GSE93070	1833	Biological interesting molecule	ENSG00000080166	13	94436808	94479682	-1	dopachrome tautomerase [Source:HGNC Symbol;Acc:HGNC:2709]
HRNR	mRNA	GSE93070	1833	Biological interesting molecule	ENSG00000197915	1	152212082	152224193	-1	hornerin [Source:HGNC Symbol;Acc:HGNC:20846]
CACNB2	mRNA	GSE93070	1833	Biological interesting molecule	ENSG00000165995	10	18140677	18543557	1	calcium voltage-gated channel auxiliary subunit beta 2 [Source:HGNC Symbol;Acc:HGNC:1402]
AP2S1	mRNA	GSE93070	1833	Biological interesting molecule	ENSG00000042753	19	46838136	46850992	-1	adaptor related protein complex 2 sigma 1 subunit [Source:HGNC Symbol;Acc:HGNC:565]
DOK7	mRNA	GSE93070	1833	Biological interesting molecule	ENSG00000175920	4	3463311	3501473	1	docking protein 7 [Source:HGNC Symbol;Acc:HGNC:26594]
PKN2	mRNA	GSE93070	1833	Biological interesting molecule	ENSG00000065243	1	88684222	88836255	1	protein kinase N2 [Source:HGNC Symbol;Acc:HGNC:9406]
TPP2	mRNA	GSE93070	1833	Biological interesting molecule	ENSG00000134900	13	102597003	102679958	1	tripeptidyl peptidase 2 [Source:HGNC Symbol;Acc:HGNC:12016]
ABCB1	mRNA	pubmed	MCF7	Biological interesting molecule	ENSG00000085563	7	87503633	87713323	-1	ATP binding cassette subfamily B member 1 [Source:HGNC Symbol;Acc:HGNC:40]
BIRC5	mRNA	pubmed		Potential biomarker	ENSG00000089685	17	78214186	78225636	1	baculoviral IAP repeat containing 5 [Source:HGNC Symbol;Acc:HGNC:593]
