# Supplementary material for: Identification of an exosome-related signature associated with prognosis and immune infiltration in breast cancer
Source: Sci Rep. 2023 Oct 24;13:18198. doi: 10.1038/s41598-023-45325-7 (PMC10598067; doi:10.1038/s41598-023-45325-7)
Supplement: Supplementary file 2 — Supplementary Table S2. [file 41598_2023_45325_MOESM2_ESM.pdf]

The primers

	FORWARD	REVERSE
**EPCAM**	5'-TTGCCGCAGCTCAGGAAGAATG-3'	5'-CGCTCTCATCGCAGTCAGGATC-3'
**PIGR**	5'-CATCCCTCCCTCCCGTCCTTC-3'	5'-TGTGAGCAGTGTTGGTGGCATAC-3'
**KRT14**	5'-TCATCCAGAGATGTGACCTCC-3'	5'-GCCTCAGTTCTTGGTGCGAA-3'
**DOK7**	5'-AGTGGAAGAGTAGGTGGCTGGTG-3'	5'-GATACGCTCCGACTTGTCCTTGTAG-3'
**CD24**	5'-GCTCCTACCCACGCAGATTTATTCC-3'	5'-CACGAAGAGACTGGCTGTTGACTG-3'
**CYP19A1**	5'-TCCATCCTTGCCAATAGTGTCATCC-3'	5'-CTTGTAGCCTGGTTCTCTGGTGTG-3'
**CXCL13**	5'-CTCTGCTTCTCATGCTGCTGGTC-3'	5'-GCTCTCTTGGACACATCTACACCTC-3'
**β-ACTIN**	5'-CATGTACGTTGCTATCCAGGC-3'	5'-CTCCTTAATGTCACGCACGAT-3'
