# Supplementary material for: Identification of an exosome-related signature associated with prognosis and immune infiltration in breast cancer
Source: Sci Rep. 2023 Oct 24;13:18198. doi: 10.1038/s41598-023-45325-7 (PMC10598067; doi:10.1038/s41598-023-45325-7)
Supplement: Supplementary file 3 — Supplementary Information 3. [file 41598_2023_45325_MOESM3_ESM.docx]

**Supporting information**

**Table S1** 121 exosome-related genes

**Table S2** The primers


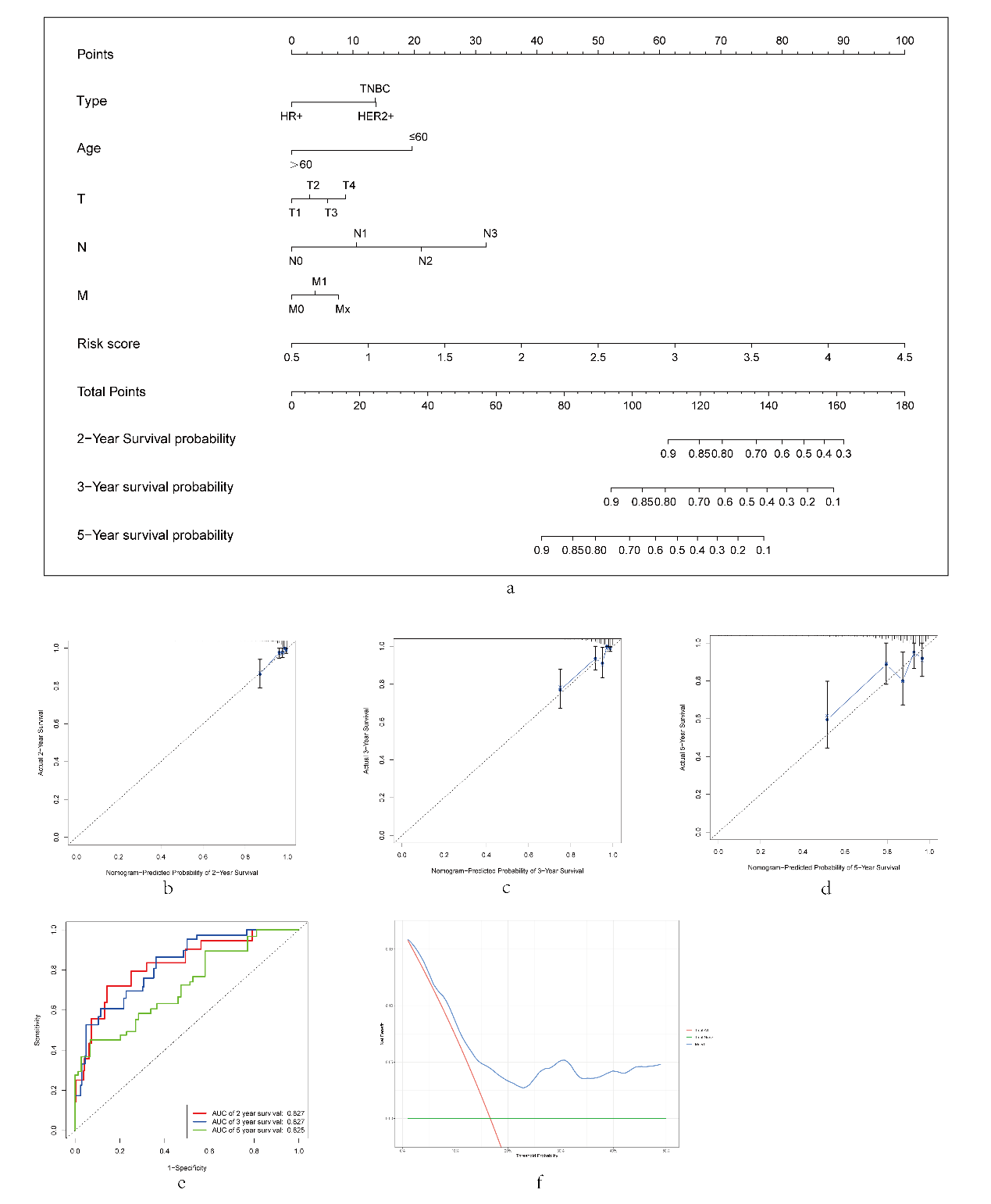


**Fig. S1** Clinical prognostic nomogram for survival prediction. (a) Clinical prognostic nomogram was applied to predict 2-, 3-, and 5-year survival by age, tumor size, lymph node status, distance metastases and risk score. Calibration curves showing nomogram predictions for 2-year (b), 3-year (c), and 5-year (d) survival. (e) Time-dependent ROC curve analysis for predicting OS at 2-, 3-, and 5-year survival. (f) The decision curve analysis of nomogram model in BC patients.


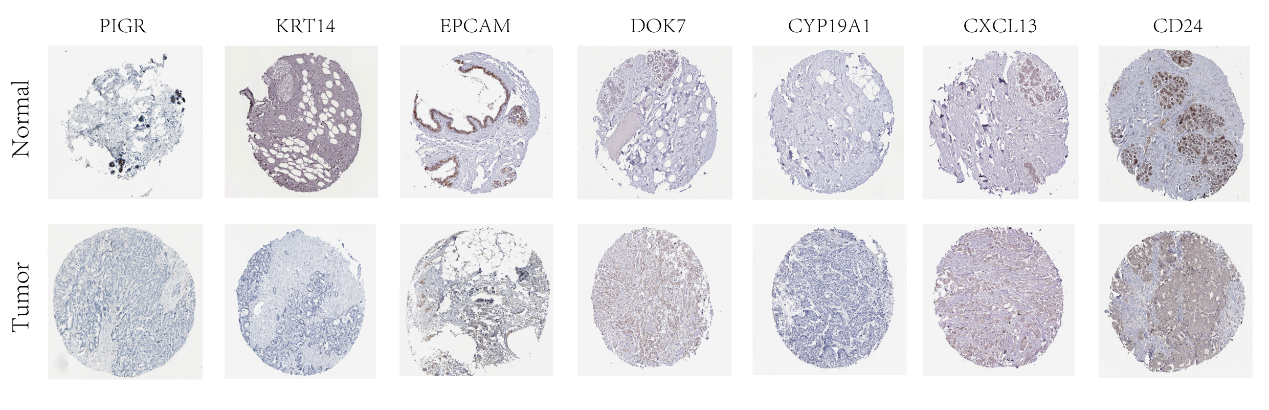


**Fig. S2** Immunohistochemical staining analysis of 7 exosome-related genes in BC based on the Human Protein Atlas.
